# Error correction enables use of Oxford Nanopore technology for reference-free transcriptome analysis

**DOI:** 10.1038/s41467-020-20340-8

**Published:** 2021-01-04

**Authors:** Kristoffer Sahlin, Botond Sipos, Phillip L. James, Paul Medvedev

**Affiliations:** 1 Department of Mathematics, Science for Life Laboratory, Stockholm University, 106 91 Stockholm, Sweden; 2 Oxford Nanopore Technologies Ltd, Oxford, UK; 3 Department of Computer Science and Engineering, The Pennsylvania State University, University Park, PA USA; 4 Department of Biochemistry and Molecular Biology, The Pennsylvania State University, University Park, PA USA; 5 Center for Computational Biology and Bioinformatics, The Pennsylvania State University, University Park, PA USA

**Keywords:** RNA sequencing, Data processing, Transcriptomics

## Abstract

Oxford Nanopore (ONT) is a leading long-read technology which has been revolutionizing transcriptome analysis through its capacity to sequence the majority of transcripts from end-to-end. This has greatly increased our ability to study the diversity of transcription mechanisms such as transcription initiation, termination, and alternative splicing. However, ONT still suffers from high error rates which have thus far limited its scope to reference-based analyses. When a reference is not available or is not a viable option due to reference-bias, error correction is a crucial step towards the reconstruction of the sequenced transcripts and downstream sequence analysis of transcripts. In this paper, we present a novel computational method to error correct ONT cDNA sequencing data, called isONcorrect. IsONcorrect is able to jointly use all isoforms from a gene during error correction, thereby allowing it to correct reads at low sequencing depths. We are able to obtain a median accuracy of 98.9–99.6%, demonstrating the feasibility of applying cost-effective cDNA full transcript length sequencing for reference-free transcriptome analysis.

## Abstract (web summary)

Nanopore sequencing technologies applied to transcriptome analysis suffer from high error rates, limiting them largely to reference-based analyses. Here, the authors develop a computational error correction method for transcriptome analysis that reduces the median error rate from ~7% to ~1%.

## Introduction

The sequencing of the transcriptome using long reads has proven to be a powerful method for understanding the transcriptional landscape of a cell^1–3^. Long-read technologies allow sequencing most transcripts end-to-end, thus overcoming the complex transcriptome assembly step required with short reads^4,5^. In particular, the Oxford Nanopore (ONT) platform is a leading technology for long-read transcriptome sequencing, due to its portability, low cost, and high throughput^6,7^. It has enabled the study of alternative splicing patterns^8^, allele-specific typing^3^, RNA modifications^6,9,10^, the discovery of novel isoforms^6,11,12^, and species identification in metatranscriptomic samples^13^.

However, the scope of ONT transcriptome studies to date has been limited because of its relatively high error rate—about 14% for both direct RNA and cDNA sequencing^11^. The most common approach to overcome this limitation is to align the reads against a reference transcriptome (e.g. GENCODE for human) or genome^11,14^. This makes the technology of limited use when a high-quality reference is not available, ruling out many non-model organisms. In addition, even when a reference is available, it does not usually capture sequence differences between individuals, cells, or environments, causing misalignment of reads from missing or highly variable loci. This has been shown to be particularly problematic in complex gene families, where a reference does not capture the high sequence diversity between individuals^15^. There are several experimental approaches to reducing the error rate^3,16,17^, but these typically come at a cost of decreased throughput and experimental overhead.

Computational error correction, on the other hand, is a highly promising approach to reduce error rates without affecting throughput or the need to customize experimental protocols. There are tools designed to correct errors in genomic reads^18–22^. But, transcriptomic error correction is challenging and differs from the genomic case because of structural variability within reads from the same gene or gene-family locus and because of highly variable and region-specific coverage within reads due to, e.g., alternative splicing, variable transcription start and end sites, and variable transcript abundances. In fact, a recent study found that applying error correctors designed for genomic reads to ONT transcriptome data had undesirable downstream effects, such as altering the isoform landscape by omitting or adding exons through overcorrection or by splitting reads at low coverage sites^23^. To achieve the potential of error correction on ONT transcriptomic data, custom algorithms have to be designed. Recent papers have tackled clustering^24,25^ and orientation problems for these data^26^ but there is currently no tool available for error correction of ONT transcriptomic reads.

In this paper, we present a method for error correction transcriptome cDNA ONT data that reduce the error rate to about 1%, thereby demonstrating the feasibility of applying cost-effective cDNA full transcript length sequencing for reference-free transcriptome analysis. We are able to achieve these error rates through a novel computational error correction method called isONcorrect, which leverages the sequence regions shared between reads originating from distinct isoforms. IsONcorrect is available for download at https://github.com/ksahlin/isONcorrect. We evaluate the method using Drosophila cDNA data generated using a modified stranded PCS109 protocol, PCS109 spike-in (SIRV) data, a human PCS108 cDNA dataset, and in silico data. Our method opens the door for much broader application of ONT transcriptome sequencing.

## Results

We will present an overview of our algorithm followed by its evaluation. For evaluation, we used one biological, one synthetic, and two simulated datasets (Table 1) to investigate the effects of error correction on read quality, error type, and splice site accuracy. We also measured the effect of read depth and parameters on the correction algorithm’s accuracy and runtime and memory usage. We present the results in this section and refer the reader to the “Experimental” and “Data analysis” sections for the relevant respective details.

**Table 1:** Datasets used in evaluation of the transcriptomic Oxford Nanopore Sequencing datasets.

Dataset	Sequencing chemistry/kit)	# unique transcripts	# reads	# inferred full-length reads (over 50nt)	Median length of full-length reads (nt)
SIM-full (chr6)	Simulated	10,367	3,500,000	3,500,000	1035
SIM-ca (chr6)	Simulated	10,367	57,804	57,804	1042
SIRV	ONT R9/PCS109	68	1,680,000	1,514,274	540
Drosophila	ONT R10/PCS109	NA	4,350,977	3,646,342	559
ONT-old	ONT R9/PCS108	NA	890,503	890,503^a^	695

^a^Dataset has been preprocessed in ref. ^11^.

### Algorithm overview

The input to our algorithm is a cluster of reads originating from transcripts of a single gene family. Such clusters can be generated from a whole-transcriptome dataset by using our previously published tool isONclust^24^. Each cluster is then processed individually and in parallel with isONcorrect, with the goal of correcting all the sequencing errors. The challenge that makes this problem different from error correction of genomic data is the highly uneven coverage within different regions of the read and structural differences between similar reads, such as exon differences and variable transcription start and stop sites.

IsONcorrect works in two stages: first, partition each read into intervals and, second, error correct each interval separately. For the first stage, we start by identifying anchor *k*-mers in each read; we use anchors as a way to identify similar sequences across reads without doing alignment of whole reads. We found alignment of whole reads is unreliable due to the compounded difficulties of having to span splice junctions, noisy reads, and variability in coverage. Technically, we use minimizers^27^ as the anchors; intuitively, it means that the chosen anchors are not too many but they are also not too far apart, guaranteeing that reads that share homologous sequence will likely also share the anchors in it.

After identifying anchors, we partition each read into a set of intervals with the following constraints: (1) each interval must begin and end at an anchor, (2) each interval’s length must be above some predefined minimum and below some predefined maximum, (3) the intervals must be non-overlapping, (4) the intervals should cover as much of the read as possible, and (5) the substring of each interval is found in as many other reads in the cluster as possible. This strategy is designed to make the computational problem tractable (conditions 1–3), to maximize the number of errors we correct during the second stage (condition 4), and to maximize our power during the second stage (condition 5). We note that condition 5 makes it unlikely that an interval crosses an exon/intron boundary, since such an interval would be found in less reads than either of the split intervals.

In the second stage of isONcorrect, we take each read and correct each interval in its partition one at a time. We identify the interval’s flanking anchors and pull out all other read substrings that are flanked by the same anchors. All these substrings are then aligned to each other to generate an alignment matrix; for each column in the matrix, we identify values that have high enough support and flag them as trusted variants. We correct the read by choosing the trusted variant whose surrounding sequence (i.e. the *k* nucleotides around it) has the smallest edit distance to the equivalent region in the read.

Please see the “Methods” section for a more detailed description of our algorithm, including some heuristic modifications not discussed here.

### Error rate analysis

**Fig. 1:**
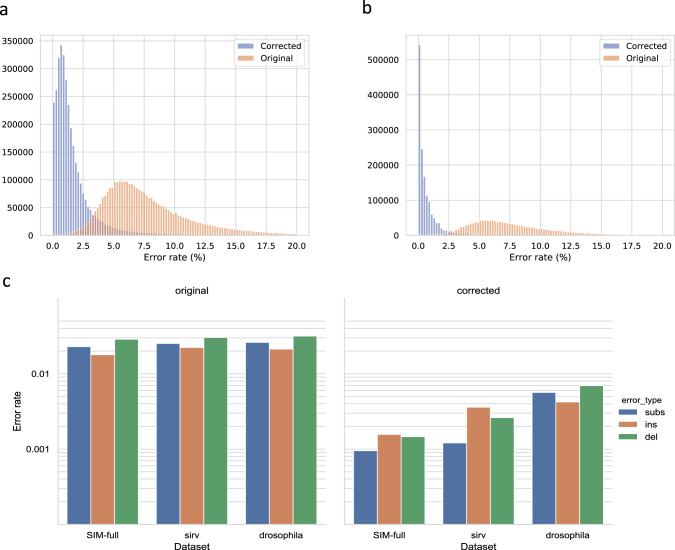
Error rates of ONT reads before and after error correction. **a** Alignment difference distribution of corrected and original Drosophila reads. Differences can arise both from sequencing errors and variation to the reference genome. **b** Error rate distribution of corrected and original SIRV reads, for the whole SIRV dataset. **c** Error profiles of the datasets before and after correction (error rate), shown on a log scale. The error rate is derived from summing up the total number of respective errors divided by the total aligned length. For Drosophila, the difference to genome is treated as an error rate in this panel.

We sequenced the transcriptome of a Drosophila sample using ONT, with a total of 4,350,977 reads (Table 1). From these, we ran pychopper and identified 3,646,342 reads as being end-to-end (which we call full length) and at least 50nt long. These reads had a median length of 559nt, and we processed the reads further by and error correcting them with isONcorrect. To measure the error rate before and after correction, we aligned the reads to the Drosophila reference genome (assembly BDGP6.22) using the spliced mode of minimap2 and counted the number of mismatches (defined as any insertion, deletion, or substitution in the alignment). We compute the error rate as the number of mismatches divided by alignment length. Errors in the reads are reflected by mismatches in the alignment; however, mismatches may also result from true biological variation in the sample and from alignment errors or artifacts. Nevertheless, we expect the mismatch numbers to be a reasonable proxy for the relative improvement in error rates. Results for before and after error correction with isONcorrect are shown in Fig. 1a. The mismatch rate decreased from a median of 7.0% to a median of 1.1% (Table 2).

**Table 2:** Read statistics before and after error correction.

Dataset	Uncorrected full-length reads		isONcorrect corrected full-length reads			RATTLE corrected full-length reads		
	#aligned	Median diff to ref (%)	#aligned	Median diff to ref (%)	# reads with more errors after correction	#aligned	Median diff to ref (%)	# reads with more errors after correction
SIM-full	3,500,000	7.0	3,500,000	0.4	1578	–	–	–
SIM-ca	57,804	7.0	57,804	0.6	214	57,804	0.4	6541
SIRV	1,512,325	6.9	1,512,450	0.4	2295	1,512,929	1.1	488,593
Drosophila	3,327,355	7.0	3,369,876	1.1	11,369	3,395,958	0.9	52,125
ONT-old	874,531	13.4	879,188	3.4	21,441	875,346	3.1	13,233

Due to the confounding of sequencing error with biological variation, we also generated a simulated dataset. We extracted 10,367 distinct transcripts from the ENSEMBL annotation of human chromosome 6 and simulated 3.5 million full-length reads from transcripts at controlled relative abundances (in the range of 1–100) from transcripts (Table 1) (for details of the simulations, see Supplementary Note 1). We denote this dataset SIM-full. Since sequencing errors were annotated as part of the simulated sequencing process, we could measure the error rate directly. As with real Drosophila data, we found that isONcorrect significantly reduces errors, with the median error rate decreasing from 7.0 to 0.4% (Table 1). Full error rate distributions for before and after error correction with isONcorrect are shown in Supplementary Fig. 1. We also studied how the post-correction error rate was affected by repetitive regions within the transcripts in the SIM-full dataset (Supplementary Note 2). We did not observe clear dependence of the average repetitiveness of the transcript sequence and the post-correction error rate (Supplementary Fig. 2).

Unfortunately, while eliminating the effect of biological variability on error rate measurement, simulated data do not always capture the full scope of errors and biases present in the real data. We therefore also evaluated isONcorrect on SIRV E0 (Spike-in RNA Variant Control Mixes) data. Our SIRV dataset consists of 68 synthetic transcripts from 7 different loci sequenced with ONT R9 technology (see section “Experimental” for details). The transcripts from each locus differ in their splicing pattern but not in any other mutation. With the SIRV dataset, we have the properties of real sequencing errors and eliminate the confounding effect of biological variation on measuring error rate. The downside of SIRV is that it does not represent the mutational complexity of a real genome. With these caveats in mind, we measured the error rate by aligning the reads to the sequences of the 68 true transcripts using minimap2 and assuming that any alignment mismatch is due to an error (see section “Data analysis” for details). Results for before and after error correction on the full SIRV dataset with isONcorrect are shown in Fig. 1b. The median error rate was 6.9% before error correction and 0.4% after (Table 2), a significant reduction.

### Error profiles

We also investigated the error profiles of the current ONT cDNA datasets before and after correction. The SIRV dataset enabled us to measure the profile of sequencing errors without the confounding effect of biological variations. We note that the overall error rate prior to correction (about 7%, Table 2) was lower than previously published cDNA ONT datasets (about 13–14%, Table 2 (ref. ^11^), likely due to improvements in experimental protocol and the basecalling software. The substitution, insertion, and deletion error rate was 2.5%, 2.2%, and 3.0%, respectively (Fig. 1c). We observed a similar distribution for Drosophila (2.6, 2.1, and 3.2%), with the caveat that it also includes true biological variation (Fig. 1c). Error correction substantially reduced the error rate in each category. The substitution, insertion, and deletion rates of SIRV reads fell to 0.1%, 0.4%, and 0.3%, respectively, after correction (Fig. 1c).

### Effect of read depth

**Fig. 2:**
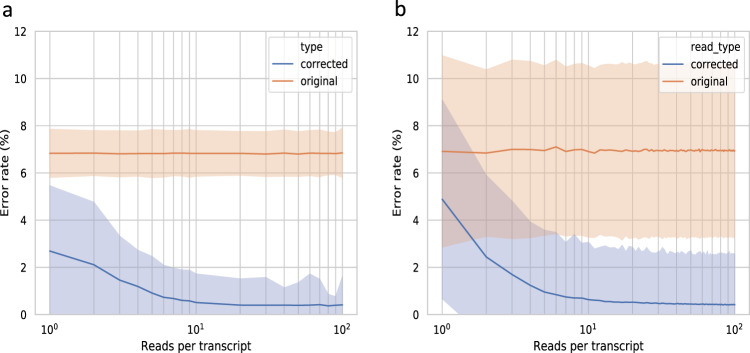
Effect of read depth on error rate. **a** shows the median error rate of the SIM-ca read experiment based on true read depth of the transcript (i.e. number of reads sequenced from it). **b** shows the median error rate of the SIRV data as a function of read depth, obtained via subsampling (see “Data analysis”). The shaded areas show the standard deviation (SD) of the error rates.

The amount of reads generated from a transcript (i.e. its read depth or, simply, depth) is typically an important factor in determining whether a tool can correct the errors in the read. To explore this in isONcorrect, we simulated a dataset with a controlled read depth for each of the 10,367 distinct chr6 transcripts (Table 1). For details of the simulations, see Supplementary Note 1. We call this dataset SIM-ca. As expected, the post-correction error rate decreased as a function of depth (Fig. 2a). Compared to the median pre-correction error rate of about 6.95%, the median post-correction error rate ranged from about 3% for depth of 1, 2% for depth of 2–3, and 0.5% for depths of 10 or more. Next, we looked at the SIRV data. Since the SIRV dataset has very high coverage, we used a subsampling strategy to investigate the error rate per sampled transcript depth (see details in the section “Data analysis”). The error rate decreased consistently for read depth up to 10, but did not improve much for larger read depths (Fig. 2b).

We note that isONcorrect remains very effective at low read depths, i.e. for read depth one, the error rate is already reduced from 7% down to 5% in SIRV and to 3% in simulated data. This is due to isONcorrect’s ability to jointly use all isoforms from a gene or gene-family during error correction, which combines information across all the transcripts with shared or similar exons. For example, the SIRV data has seven gene loci with several splice variants each (between 6 and 18), meaning that each exon will have higher coverage than any individual transcript. Transcripts occurring at very low read depths that have exons with unique mutations or small variations in splice junctions may however be overcorrected. We investigate such artifacts in the following sections.

### Splice site accuracy and transcript recovery

One of the potential benefits of error correction is obtaining nucleotide-level resolution of splice sites. Simultaneously, correction around borders of splice junctions is known to be challenging and may alter the splice site, particularly if it is present only at low abundances^23^. Since the Drosophila reference genome has high-quality gene annotations, we used alignments to classify each read according to how it matches the annotated splice sites, using the terminology of ref. ^28^ (see “Data analysis”).

**Fig. 3:**
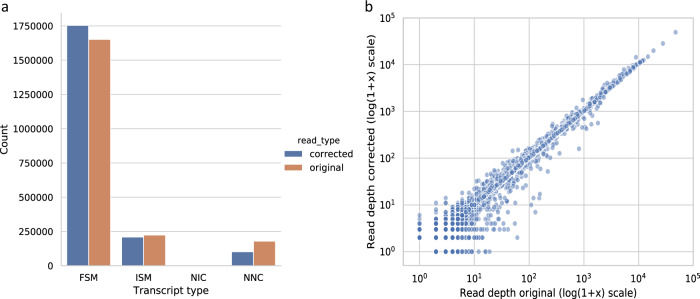
Splice site accuracy before and after error correction in the Drosophila data. **a** Total number of reads classified per splice site category, using the terminology of ref. ^28^. FSM full splice match, ISM incomplete splice match, NIC novel-in-catalog, NNC novel-not-in-catalog. **b** For each transcript in the reference, we measure the number of reads aligning to it as an FSM, before and after error correction. Each dot represents a distinct transcript with at least one FSM in either the original or corrected reads.

As expected, we observed more reads fully matching an annotated transcript (FSM, full splice match) after correction (Fig. 3a). We did not see any novel combinations of splice sites (NIC, novel-in-catalog) in the reads before or after correction. This is not surprising given the high-quality annotation of the Drosophila genome. However, it did underscore a positive aspect of ONT sequencing, which is that no artificial transcripts have been constructed in the experimental steps of generating the data, such as reverse transcriptase template switching.

We did observe slightly more reference transcripts that have at least one FSM read in the original reads compared to corrected reads (13,062 and 12,982, respectively, with 178 lost and 99 gained) and investigated the lost transcripts after correction as a function of how abundant they were in the original reads (Fig. 3b). Out of the 178 transcripts that were not captured by an FSM read after correction, 107 and 37 of them had only one and two FSM original reads, respectively, and all but 4 of them occurred in less than 10 original reads. Therefore, a consequence of our correction algorithm is that the lowest abundant transcripts may be miscorrected. However, we also observed 99 transcripts had no FSM support before correction but did after error correction. As the error correction is reference agnostic, this is likely due to reads from annotated transcripts that were misaligned around splice sites prior to correction, and highlights the benefit of reference-free error correction.

### Overcorrection

One pitfall of using an alignment-based evaluation method is when the error correction algorithm modifies non-erroneous positions in a way that the read more closely aligns to the reference genome. For example, such overcorrection can happen both because of allele-specific or gene-copy-specific variation that produce similar transcripts differing in only single-nucleotide polymorphisms (SNPs). More formally, we classified a simulated read as overcorrected if the read has an edit distance smaller to a transcript other than the true transcript. This is computed by first aligning reads with minimap2, and then comparing the edit distance of minimap2’s primary alignments to the edit distance to the true transcript. Such overcorrection is an undesirable artifact because it misrepresents the biological sample; however, when using an alignment-based evaluation method, overcorrection can go undetected because it can actually improve the inferred error rate. Nevertheless, we were able to measure the presence of overcorrection using our SIM-ca simulated dataset, where the true transcript is known.

**Fig. 4:**
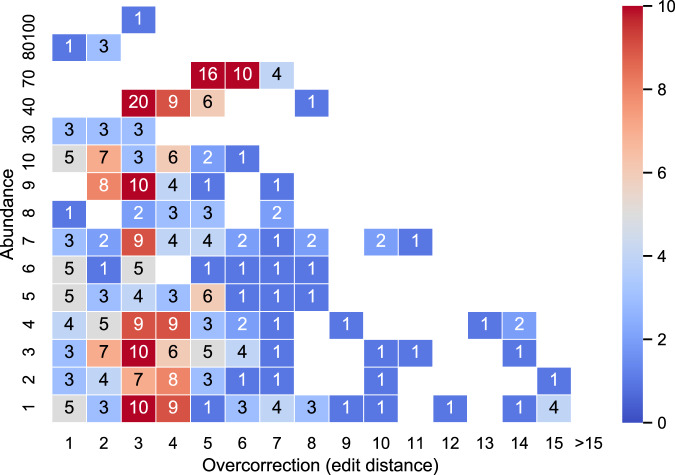
The effect of overcorrection in SIM-ca. We bin each overcorrected read according to the abundance of its true transcript (*y*-axis) and its overcorrection distance (*x*-axis). Each cell shows the number of reads in the bin.

The overcorrected reads made up less than 0.6% of all reads (359 out of 59,440; Fig. 4). Note that a small fraction of the reads, particularly from highly similar transcripts, may be included in our definition of overcorrected because initial sequencing errors made them more similar to another transcript than the original one; these are really instances of not enough correction rather than overcorrection. We observed this in 76 original reads (21% of overcorrected reads), i.e. reads that aligned better to a transcript different to the true transcript even before error correction.

To investigate further, we measured how much closer the overcorrected reads were to the incorrect transcript. We computed the overcorrection distance for a read as the edit distance of the read to its true transcript minus the edit distance to its closest aligned transcript. We then plotted the overcorrection distance together with the abundance of the true transcript, for the overcorrected reads (Fig. 4). We found that this distance was small for the vast majority of isONcorrect’s overcorrected reads, i.e. 5 or less positions in >80% of the overcorrected reads. In addition, the overcorrection was mostly limited to reads at low abundances, with 47% of overcorrected reads coming from transcripts with an abundance of ≤5. This indicates that overcorrection with isONcorrect is mostly limited to SNPs or short indels, as opposed to larger exon-level miscorrections.

### Effect of pre-correction error rate

Since the error rate of the sequencing technology can change over time, we investigated how it would affect the performance of isONcorrect. Our SIM-ca dataset had a median pre-correction error rate of 7% and we will refer to it as SIM-ca-7 in this section. We also generate datasets SIM-ca-4 and SIM-ca-11 which had median pre-correction error rates of ~3.94% and ~11.40%, respectively (Supplementary Note 1).

The post-correction error rates, as a function of depth, are shown in Fig. 2a for SIM-ca7, Supplementary Fig. 3a for SIM-ca-4, and Supplementary Fig. 3b for SIM-ca-11. The overall post-correction median error rate was 0.4%, 0.6%, and 1.7% for SIM-ca-4, SIM-ca-7, and SIM-ca-11, respectively. We observe in these figures that the tails are longer for higher pre-correction error rates, i.e. for higher pre-correction error rates, more read depth is needed to achieve a stable post-correction error rate.

We also measured how overcorrection was affected by the pre-correction error rate (Supplementary Fig. 3C, D). The percentage of overcorrected reads was 0.3% in SIM-ca-4 (Supplementary Fig. 3C), 0.6% in SIM-ca-7 (Fig. 4), and 0.8% in SIM-ca-11 (Supplementary Fig. 3D). Similar to what we previously observed in the SIM-ca-7 dataset, overcorrection in SIM-ca-4 and SIM-ca-11 was mostly limited to reads that came from transcripts with multiple similar copies and that occurred at relatively low abundances (Supplementary Fig. 3C, D).

Overall, we conclude that isONcorrect behaves as expected; i.e. the post-correction error rate and the number of overcorrected reads are lower when the pre-correction error rate is lower.

### isONcorrect on an older ONT protocol

We also investigated isONcorrect’s performance on a dataset from an older cDNA protocol (denoted ONT-old; Table 1) of human ONT cDNA sequencing with a minION^11^. This dataset has a pre-correction error rate of 13.4%. On this dataset, isONcorrect is able to reduce the error rate to 3.4%, increase the number of aligned reads (Table 2 and Supplementary Fig. 4A), and increase the number of FSM reads (Supplementary Fig. 4B). The 3.4% post-correction error rate is due to the higher pre-correction error rate and is consistent with our analysis on simulated datasets. For example, in the SIM-ca-7 and SIM-ca-11 datasets, a 63% (11.4/7.0) increase in pre-correction error rate leads to a 183% (1.7/0.6) increase in post-correction error rate. In comparison, in the two biological datasets Drosophila and ONT-old, we have a 91% (13.4/7.0) increase in pre-correction error rate, which leads to a 209% (3.4/1.1) increase in post-correction error rate. However, we do not exclude the possibility that the complexity of the transcriptome can be a contributing factor to this increased post-correction error rate.

On this dataset, in contrast to the *Drosophila* dataset, we observed more reference transcripts that have at least one FSM read in the corrected reads compared to the original reads (7788 and 7710, respectively, with 377 lost and 462 gained). We investigated the lost transcripts after correction as a function of how abundant they were in the original reads (Supplementary Fig. 4C). Out of the 377 transcripts that were not captured by an FSM read after correction, 299 and 44 of them had only one and two FSM original reads, respectively, and all but six of them occurred in less than 10 original reads. Similarly to what we observed in the *Drosophila* dataset we observe that the lowest abundant transcripts may be miscorrected.

### Effect of number of isoforms

Using the SIRV data we also investigated how the post-correction error rate changed with different numbers of isoforms per gene and with read depth. We performed subsampling of reads from three gene loci in the SIRV data that had at least eight isoforms each (SIRV3, SIRV5, and SIRV6). Our subsampling was performed as in the read depth experiments (see details in “Data analysis” section under “SIRV subsampling experiments”).

We found that the same post-correction error rate was achieved regardless of the number of isoforms (Supplementary Fig. 5). However, less reads per isoform were needed to achieve equivalent error rates for experiments with more isoforms. This may seem counterintuitive but is due to isONcorrect’s ability to combine reads from different isoforms for the purposes of error correction. For example, a median error rate of 2% is reached after 3, 4, and 5 reads per isoform in the datasets with 8, 4, and 1 isoforms, respectively.

We also observe that while higher numbers of isoforms do not affect the post-correction error rate, they do result in a higher fraction of overcorrected reads (Supplementary Fig. 6). When we aggregate the overcorrection percentages for experiments that had the same number of isoforms, they are 2.7%, 1.6%, and 0.3% for the experiments with eight, four, and one isoforms, respectively. Increased read depth does not help to alleviate the problem, because the relative depth of the confounding isoforms does not change (Supplementary Fig. 6). However, we note that the overcorrection is again mostly due to small variations rather than exon-level changes; for example, we observed that 57% of the overcorrected reads (across all experiments) switched isoforms between SIRV506 and SIRV511, which are identical except for a splice junction difference of 6 nt.

### Overcorrection of SNPs with allele-specific expression

To study how well isONcorrect can distinguish errors from allele-specific SNPs, especially when the alleles have different expression levels, we simulated data reads from two transcripts in an isolated setting. We started with two identical copies of a transcript from the RTN4IP1 gene of length 208nt. In each experiment, we inserted an SNP in one of the copies at a randomly chosen position (between 50 and 150nt). We then simulated a given number of reads (*n*) (at a 7% error rate as in our other simulations), with a given fraction (*f*) coming from the mutated transcript. We varied the number of reads *n* from 10 to 100 and the relative frequency of the SNP (*f*) between 10 and 50%. For each *n* and *f*, we ran 10 replicate experiments. We then measured the fraction of replicates which had at least one SNP-containing read after error correction. Our experimental design is intended to vary the sequence context of the SNP and the locations of the errors while controlling for read depth and SNP frequency.

Figure S7 shows the result. At least 20 reads are needed to retain SNPs at 20 or 30% frequency with a probability of 80%. For SNPs at 10% frequency, at least 50 reads are needed. This corresponds to about 4–6 reads needed to cover the minor SNP allele in order to avoid overcorrecting it.

IsONcorrect’s behavior is guided by a formula which is a function of the number of reads coming from the minor allele and the sequence context around the SNP (see “Algorithmic details”). Intuitively, in the simple case of an isolated SNP with no sequencing errors within *k/2* nucleotides, the formula will retain the SNP if its frequency is at least 10% and there are at least three reads covering it. When there are multiple SNPs nearby, this threshold decreases, while when there are sequencing errors nearby, this threshold increases.

### Overcorrection of exons

We study how error correction with isONcorrect preserves the exon structure for transcripts at different abundances, using both simulated and SIRV data. For the simulated data, we used a similar design to the allele-specific expression experiment. We started with two identical copies of a transcript from the RTN4IP1 gene. In each experiment, we removed a substring (of length 5, 10, or 20nt) in one of the copies at a randomly chosen position (between 50 and 150nt). We chose short length substrings because shorter exons pose a bigger challenge to isONcorrect than longer ones. We then simulated a given number of reads (*n*) (at a 7% error rate as in our other simulations), with a given fraction (*f*) coming from one of the two transcripts (chosen at random). We varied the number of reads *n* from 10 to 100 and the relative frequency of the minor isoform (*f*) between 10 and 50%. For each *n* and *f*, we ran 10 replicate experiments. We then report the fraction reads with best alignment to the minor isoform before and after correction (Fig. S8). We observe that substantial overcorrection occurs when the exon is very small (5nt), at all read depths and minor isoform fractions. However, no noticeable overcorrection occurs at 20 bp exons, and the overcorrection for 10bp exons is negligible for most downstream analyses.

For the SIRV data, we took the only two available isoform pairs, SIRV506 and SIRV511, and SIRV606 and SIRV616, that are identical besides differing in an internal splice site. This internal splice difference gives rise to a 6 and 14nt deletion, respectively, between two otherwise identical transcripts. We then used the subset of the SIRV reads that had primary alignments (using minimap2) to each of the four transcripts, and furthermore, an edit distance strictly smaller to one of the transcripts. The edit distance constraint was invoked because some reads were ambiguous to which transcript they belonged, particularly for the transcript pair with 6nt distance. We then subsampled reads from the subset of reads from each isoform at different controlled depths and fractions and computed the overcorrection in the same manner as for the simulated dataset. As for the simulated dataset we report the fraction reads with best alignment to the minor isoform before and after correction (Supplementary Fig. 9). In the case of the 6nt deletion, we observe substantial overcorrection at low (≤20 reads) read depths. For the 14nt deletion, we observe overcorrection similar to the case of 5nt in the simulated experiment. The change in isoform fraction is up to 10–20 percentage points. The reason for the difference between SIRV data and our simulated results may be due to the sequence of the transcript (and, in particular, the deleted exon is fixed compared to simulated data, where it is randomly varied across experiments), the ONT-specific error profile, selection of reads in the experiment, or to other unknown causes. In summary, we observe that while exon overcorrection is in general more frequent in our SIRV data than on the simulated data, substantial overcorrection occurs when the exon is small and read depth is low for the minor isoform (Supplementary Fig. 9A).

### Effect of heuristics and parameters

For large clusters, isONcorrect uses a heuristic approximate algorithm (see “Methods”). While this reduces the runtime, it has the potential to reduce the quality of the results. We therefore investigated the accuracy between the approximate and exact mode using controlled subsampled reads from the SIRV dataset (see “Data analysis” section for details). As expected, we observed a decrease in accuracy in approximate mode compared to exact mode across all different *k* and *w*, with the difference in accuracy decreasing as read depth increases (Supplementary Fig. 10). However, the accuracy differences between the two modes were negligible compared to the improvements over the uncorrected reads.

We also investigated the effect of parameter choices for the *k*-mer size *k*, and window size *w*, and the maximum anchor distance *x*_max_. We observed minor effects across different *k* and *w* (Supplementary Fig. 10). However, isONcorrect performs well over all the tested values of *k* and *w*, with the difference being minor compared to the overall effect of correction and of the read depth Overall, we obtained slightly better results for *k* = 9 which we set as the default value to isONcorrect. As for the maximum anchor distance, we saw a minor improvement in longer spans (80–100) compared to 40 (Supplementary Fig. 11), and this informed us to set default value of *x*_max_ = 80. We generally conclude, however, that parameter values within the tested ranges have only a minor effect on accuracy.

### Runtime and memory

We measured runtime and memory of isONclust and isONcorrect (Table 3). We used a machine with an x86_64 system running Linux v3.2.0-4-amd64 and equipped with 32 2-threaded cores and 512 GB RAM. We allowed isONclust to use 50 threads and isONcorrect to use 62 threads. While isONclust is relatively fast, the correction with isONcorrect takes significant time (over 2 days). Given the time investment into the sequencing protocol, we consider this time expense tolerable. However, we hope to speed up isONcorrect in the future by allowing parallelization across nodes, making it possible to speed up correction by running it on a multi-node cluster.

**Table 3:** Runtime and memory usage of error correction pipelines.

Dataset	pychopper		isONclust		isONcorrect		RATTLE cluster		RATTLE correct	
	Peak memory	Runtime	Peak memory	Runtime	Peak memory	Runtime	Peak memory	Runtime	Peak memory	Runtime
Drosophila	5 Gb	32 min	34 Gb	2 h 05 min	102 Gb	43 h 54 min	48 Gb	16 h 52 min	314 Gb	1 h 34 min
SIM-ca	–	–	8 Gb	0 h 08 min	99 Gb	1 h 40 min	2 Gb	14 min	83 Gb	9 min
SIRV	3 Gb	14 min	10 Gb	0 h 9 min	127 Gb	6 h 13 min	19 Gb	28 min	18 Gb	49 min
SIM-full	–	–	49 Gb	2 h 13 min	385 Gb	54 h 49 min	108 Gb	1 h 29 min	–	–
ONT-old	–	–	24 Gb	4 h 43 min	89 Gb	11 h 11 min	14 Gb	11 h 51 min	14 Gb	23 min

RATTLE correct on SIM-full did not complete because it exceeded the 500 Gb memory capacity of our server (we tried using 1, 2, 4, 8, 16, 32, and 60 cores).

The current memory usage requires a large memory server to run. We note that in our simulated data, some transcripts were very long (>20,000 nucleotides). This resulted in a large memory consumption given the number of reads compared to the SIRV and Drosophila data. It is possible to decrease memory usage in several ways, such as increasing *w* or decreasing *x*_max_, at the potential cost of accuracy. However, the memory footprint can be greatly reduced by implementing isONcorrect in C++ or storing minimizers and paired anchors in more efficient data structures^29^.

### Comparison against other tools

As Lima et al.^23^ found that applying genomic error correctors to ONT transcriptome data had undesirable downstream effects, we only evaluated the genomic error corrector CONSENT^30^ that was not evaluated in ref. ^23^ as well as canu^18^ and concluded similarly to ref. ^23^ that they are not suitable for long transcriptomic reads (see Supplementary Note 3). We instead focused on comparing our tool to a recent long transcriptomic read error correcting tool RATTLE^31^.

We ran RATTLE on our five datasets (Table 1) and results are shown in Table 2. RATTLE showed comparable median error rates to isONcorrect on the *Drosophila*, SIRV,SIM-ca, and ONT-old datasets (0.9%, 1.1%, 0.4%, and 3.1% for RATTLE and 1.1%, 0.4%, 0.6%, and 3.4% for isONcorrect, respectively). However, we observed that a significant fraction of reads were miscorrected by RATTLE, which we define as reads that have more errors after correction than before. Specifically, the percentage of reads that were miscorrected by RATTLE were 1.5%, 32%, and 11.3% for the *Drosophila*, SIRV, and SIM-ca datasets compared to 0.3%, 0.2%, and 0.4% by isONcorrect (Table 2). For Drosophila and SIRV, these numbers may be influenced by mapping ambiguities, but for the simulated dataset they are exact. For the ONT-old dataset, we observed less reads classified as miscorrected for RATTLE (1.4%) compared to isONcorrect (2.4%).

We further studied the effect of error correction to alterations in splice structure, a negative side effect of error correction that our mis-correction measure does not explicitly capture. We used the *Drosophila* and the ONT-old data. Similarly to isONcorrect, RATTLE increased the number of reads fully matching an annotated transcript (FSM) on both Drosophila (Supplementary Fig. 12A) and ONT-old (Supplementary Fig. 12B). However, for both Drosophila and ONT-old, we observed significantly less reference transcripts having at least one RATTLE corrected FSM read (9893 and 5597, respectively) than having at least one original FSM read (13,062 and 7710, respectively). This corresponds to a 24.3% and 17.4% reduction, respectively, compared to only a 0.6% reduction and 0.1% increase for isONcorrect. Specifically, for Drosophila RATTLE lost 3248 and gained 79 transcripts with at least 1 FSM, while isONcorrect lost 180 and gained 100 transcripts. In some cases, transcripts that had as many as 756 original FSM reads were lost (Supplementary Fig. 12C). For ONT-old, RATTLE lost 2519 and gained 406 transcripts with at least 1 FSM, while isONcorrect lost 377 and gained 462 transcripts. Similarly to the Drosophila dataset, we observe a substantial alteration of splice sites in the FSM reads for the ONT-old dataset after correction with RATTLE (Supplementary Fig. 12D) compared to isONcorrect (Supplementary Fig. 4C). Our splice site analysis on the two biological datasets showed that RATTLE correction can miscorrect substantially more correct splice sites than isONcorrect.

We further investigated RATTLE’s overcorrection using the SIM-ca dataset (Supplementary Fig. 12E), in the same way we did for isONcorrect (Fig. 4). We observed that 16.7% (9947 out of 59,440) of RATTLE corrected reads aligned with better identity to a transcript other than the true transcript (compared to just 0.6% for isONcorrect). These overcorrected reads were found throughout different abundances and different magnitudes of overcorrection.

We also used the SIM-ca dataset to analyze the effect of read depth on correction accuracy of RATTLE (Supplementary Fig. 12F). We observed that RATTLE needs a depth of at least six reads to decrease the median error rate (Supplementary Fig. 12F), but the standard deviation of the median remains large even at larger depths because of the large fraction of miscorrected reads (11.3%; Table 2). IsONcorrect, on the other hand, decreases the median error rate for reads already at depth 1, with a narrower standard deviation due to significantly fewer miscorrected reads (Fig. 2a). Reducing error rate for reads at low depths is expected with isONcorrect, as it is designed to leverage shared exons, while RATTLE is not designed to make use of shared exons between different isoforms in the error correction.

As for resource utilization, RATTLE’s clustering and correction pipeline is faster than that of isONclust and isONcorrect (Table 3). However, RATTLE uses more memory (three times of isONcorrect for Drosophila) and exceeded 500 Gb on the human SIM-full dataset. It was not able to complete on SIM-full because it exceeded the available memory on the server.

## Discussion

We presented a novel computational tool isONcorrect to error correct cDNA reads from Oxford Nanopore Technologies. On a Drosophila dataset, the raw data had an initial mismatch rate of 7.0%, which isONcorrect further decreased to 1.1%. This is a drastic improvement over previously published ONT transcriptome mismatch rates of about 14%^11^. Compared to the R2C2 (Rolling Circle Amplification to Concatemeric Consensus) method, which modifies the experimental protocol, our approach does not decrease the throughput and achieves a significantly better mismatch rate (2.5% for R2C2)^2,17,32^.

Evaluating the error rate of a transcriptome read error correction tool is a challenge due to, on the one hand, the presence of biological variation and alignment ambiguity in real data, and, on the other hand, the limitations of simulated and synthetic data. In this paper, we took the kitchen sink approach and evaluated isONcorrect’s performance on all these datasets. Our results showed consistent performance (Table 2), with the resulting mismatch rates between 0.4 and 1.1%. We also observe that the correction is not always correct, with 0.6% of the reads being overcorrected, and that SNPs or short indels occurring at low relative abundance are not always retained.

One of the underlying strengths of the isONcorrect algorithm is its ability to error correct reads even if there are as little as one read per transcript. The idea is to leverage exons that are shared between different splice isoforms. To achieve this, we pre-process the reads using our isONclust clustering algorithm, which clusters reads according to the gene family of origin. This strategy is in sharp contrast to approaches which cluster based on the isoform of origin. Such clustering results in low read coverage per transcript^24^, particularly for genes expressing multiple isoforms with variable start and stop sites and makes error correction unable to utilize full coverage over shared exons. By using isONclust to cluster at the gene-family level, each read retains more complete exon coverage and helps the correction process preserve allele- or copy-specific small variant differences between transcripts that otherwise share the same structure. This effect is shown in our experiments, where there is already a significant reduction in the error rate (down to 3–5%) for transcripts with just one read. However, there is a disadvantage that one gene’s transcript that shares an exon with another gene’s transcript may get miscorrected if it occurs at a much lower frequency.

IsONcorrect relies on two additional key algorithmic components to achieve scalability and high accuracy. First, we are able to partition the reads within a cluster into exon-like segments in a way that maximizes the read depth of each segment by formulating the problem as an instance of the classical weighted interval scheduling problem. This scheduling problem can then be solved optimally using an efficient and exact dynamic programming algorithm^33^. IsONcorrect is then able to separately correct the regions produced from the scheduling solution, where each region can have highly variable coverage but the coverage within a region is roughly equal. Second, we identify heuristic optimizations that drastically speed up our algorithm and adaptively apply them when the expected runtime is expected to be slow. We show empirically that these heuristics do not significantly reduce the accuracy.

There exist other algorithms for reference-free error correction of long transcriptomic reads that are specific to the Pacific Biosciences IsoSeq platform. These include ToFU/isoseq3 (ref. ^4^) and IsoCon^15^, which perform both clustering and error correction and the final result is predicted unique transcripts. Isoseq3 is inherently limited to IsoSeq data, while IsoCon, which is intended for targeted sequencing data, assumes high exon coverage and is not designed to handle variable start/end sites, which are ubiquitous in non-targeted datasets. Other approaches use short read data for error correction of long IsoSeq reads^34,35^.

There also exist several methods for error correction of ONT genomic data, both long-read-only and hybrid (short + long reads). We do not compare against these because a recent comprehensive benchmark showed that applying these to transcriptome data is problematic^23^. While these tools reduced the error rate from about 13% down to 4%, all the tools also reduced the number of detected genes, gene-family sizes, and the number of isoforms; they also reduced the number of detected splice sites and split reads up in low coverage regions. Similar findings were also observed in ref. ^36^ for genomic error correctors applied to PacBio’s IsoSeq transcriptome reads. Given that genomic error correction tools alter the structural landscape of these reads, we do not consider them useful for most transcriptome applications.

Our strategy for partitioning of the read into separate intervals is based on a related idea used in the context of genomic error correction in the algorithm CONSENT^30^. CONSENT identifies alignment piles within reads, i.e., a set of reads aligning to the same region, where the alignments are produced by a third party genomic read aligner. Within an alignment pile, CONSENT finds the longest collinear chain of anchor *k*-mers shared by at least a threshold of the reads in the pile. The collinear regions are then used to split the pile into segments which are error corrected separately. This approach was shown to reduce runtime^30^ while solving a simpler local and hopefully less error-prone problem. However, it does not guarantee that the *k*-mer anchors are chosen so that the segmentation solution is optimal with respect to read coverage across the segments in the read. We make use of the idea of interval segmentation and anchor *k*-mers, but adapt the approach to a transcriptomic context. As structural differences and variable coverage is at the heart of transcriptomic error correction, we solve the partitioning problem by formulating it as a global (with respect to the read) *k*-mer anchor optimization problem over the anchor *k*-mer depth. In addition, we cannot rely on third party tools to find self-to-self transcriptome read alignments, as this is a challenging problem due to the variable abundance.

The protocol used in this paper is based on the sequencing of cDNA, but there also exists a ONT protocol to sequence RNA directly^7,11,37–39^. Direct RNA sequencing with ONT is a promising alternative to cDNA sequencing, but its potential has not yet been realized because of higher error rates (14%), low throughput, and the inability to guarantee reads spanning the full transcript^11^. Because of high error rates, some of the analysis in ref. ^11^, e.g. splice site analysis or allele-specific expression, was done using a combination of the GENCODE reference and the sequencing of cDNA from the same sample. On the other hand, cDNA sequencing produces high throughput and can, through experimental and computational methods, produce reads that are guaranteed to span the full molecule. With the method in this paper, the cDNA approach can now achieve error rates of about 1%, making it applicable to reference-free analysis. However, applying isONcorrect to direct RNA reads is a direction for future work that should enable the reference-free use of direct RNA reads.

## Methods

### isONcorrect algorithm

**Fig. 5:**
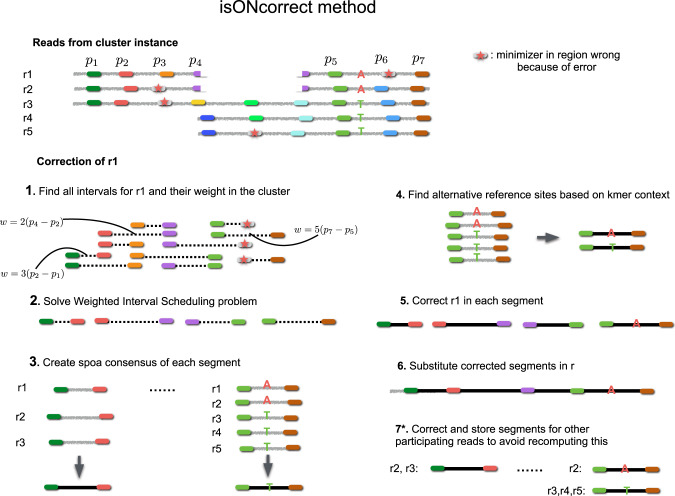
Overview of isONcorrect. The input to isONcorrect is reads from a single cluster produced by isONclust (or any other software that group reads into gene families of origin). This figure illustrates a cluster with five reads (*r*1–*r*5) from three isoforms. isONcorrect finds all intervals with distance between *x*_min_ to *x*_max_ using anchor minimizers (shown as colored blocks) and adds them to a hash table. To correct a single read (e.g. *r*1), all the anchor minimizer pairs found in *r*1 are queried in the hash table, and all reads containing a given anchor minimizer pair are retrieved. In this example, *r*1 has 11 such anchor pairs (shown in Step 1). Each anchor pair is assigned a weight that is the product of its span and the number of reads containing this anchor pair (with the exception of filtering out anchor pairs of dissimilar regions; details in “Methods”; Step 1). For example, the anchor pair (*p*1, *p*2) occurs in three reads (*r*1, *r*2, and *r*3). The instance is sent to a weighted interval scheduler that finds the set of non-overlapping anchor pairs with the largest weight (Step 2). In this case, four anchor pairs are selected. All segments between the chosen anchor pairs are sent for correction. A consensus is created (Step 3) using spoa, and one or more trusted variants are identified, based on their frequencies and sequence contexts (Step 4). Each read segment in *r*1 is corrected to the closest trusted context (Step 5). The segments are inserted back into the original read *r*1 in what becomes the corrected read of *r*1 (Step 6). An optional Step 7 corrects the segments of the other reads in the same manner and stores them in a hash table to be retrieved whenever it is their turn to be corrected. For example, when it is *r*2’s and *r*3’s turn to be corrected, the interval spanned by the anchor pair (*p*1, *p*2) may be again encountered in the optimal scheduling solution, allowing Steps 3–5 to be skipped at that point.

The “Results” section contains a high-level overview of our algorithm. Here, we describe isONcorrect in detail, by giving the relevant definitions, the steps of the algorithm, and finally the heuristic modifications to improve runtime. The steps of the algorithm are illustrated in Fig. 5.

#### Definitions

Let *r* be a string of nucleotides that we refer to as a read. We use *r*[*i*] to refer to the *i*th position of *r*. Given two integers *k* and *w* such that 1 ≤ *k* ≤ *w* ≤ |*r*|, the minimizer of *r* at position *p* is the lexicographically smallest substring *m* of length *k* that starts at a position in the interval of [*p*, *p* + *w* − *k* + 1). We then say that *r* has a minimizer *m*, or, alternatively, has a *positional minimizer* (*m*, *p*). For example, for *r* = *AGACCAT*, *k* = 2, *w* = 3, we have that the ordered set *M* = {(*m*_*i*_, *p*_*i*_)} of positional minimizers are *M* = {(*AG*, 0), (*AC*, 2), (*CA*, 4), (*AT*, 5)}. Let *x*_min_ and *x*_max_ be two positive integer parameters, where we call *x*_max_ the maximum anchor distance. Then we let *W*_*r*_ = {((*m*_*i*_, *p*_*i*_), (*m*_*j*_, *p*_*j*_)) ∈ *M* × *M* | *x*_min_ ≤ *p*_*j*_ − *p*_*i*_ ≤ *x*_max_} be the ordered set (according to increasing *p*_*i*_ than *p*_*j*_) of paired positional minimizers separated by at least *x*_min_ and at most *x*_*max*_ nucleotides in *r*. Similarly, we let *StrW*_*r*_ = {(*m*_*i*_, *m*_*j*_) | ((*m*_*i*_, *p*_*i*_), (*m*_*j*_, *p*_*j*_)) ∈ *W*_*r*_} be the sequence of paired minimizers, i.e., *W*_*r*_ with the positions omitted but duplicates retained. For example, the above set of minimizers with *x*_min_ = 2, *x*_max_ = 3 gives *W*_*r*_ = {((*AG*, 0), (*AC*, 2)), ((*AC*, 2), (*CA*, 4)), ((*AC*, 2), (*AT*, 5)), ((*CA*, 4), (*AT*, 5))} and *strW*_*r*_ = (*AG, AC*), (*AC, CA*), (*AC, AT*), (*CA, AT*). Given a set of reads *R*, we let *W* be the union of all *W*_*r*_ for the reads in *R* and we let *StrW* be the union of all *StrW*_*r*_.

The weighted interval scheduling problem takes as input a set of intervals *I* = {*i*_1_,…*i*_*n*_}, where *i*_*j*_ ∈ [*a*_*j*_,*b*_*j*_], *a*_*j*_,*b*_*j*_ ∈ *R,* and _*j*_ < *b*_*j*_, and a weight *w*_*j*_ associated with each *i*_*j*_. The output is a subset *I*′ ⊆ *I* of non-overlapping intervals whose sum of weights is maximized. The weighted interval scheduling problem can be solved exactly using a dynamic programming algorithm that runs in *O*(*n* log*n*) time, where *n* is the number of intervals^33^

We will use *ed*(·) to refer to the edit distance between the two strings, and we use edlib^40^ to calculate the edit distance. We will use *HC*(·) to denote the homopolymer compression function. Given a string *s*, *HC*(*s*) removes from *s* all characters identical to the preceding one, i.e. *HC*(*ATTTCAA*) = *ATCA*.

#### Algorithm details

For a given cluster, we first generate all the paired positional minimizers *StrW* of the reads. Then for each read *r* we will construct a weighted interval scheduling instance (Step 1). Each positional minimizer pair ((*m*_1_, *p*), (*m*_2_, *q*)) ∈ *W*_*r*_ defines an interval on *r* that is spanned by, but does not contain the minimizers, i.e [*p* + *k*, *q*). The interval is given the weight *a*(*q* − *p* − *k*), where *a* denotes the support of the interval. We compute the support as the number of occurrences of (*m*_1_, *m*_2_) in *StrW* whose intervals have a similar sequence to the one spanned by (*m*_1_, *m*_2_) in *r*, and this is computed as follows. Let *r*′ ≠ *r* be a read containing (*m*_1_, *m*_2_) with coordinates (*p*′, *q*′) in *r*′ and let *s* = *r*[*p*:*q* + *k*] and *s*′ = *r*′ [*p*′:*q*′ + *k*] be substrings of *r* and *r*′ spanned by, and including, the minimizer windows. We consider that *s*′ has similar sequence to *s* if the edit distance is less than |*s*| (ϵ_*s*_ + ϵ_*s*′_), where ϵ_*s*_ (respectively, ϵ_*s*′_) is the average per-base error rate of *s* (respectively, *s*′), inferred from the quality values. If a read has multiple (possibly disjoint) intervals matching a single interval of *r*, only the one with the smallest edit distance is considered.

Next, for the read *r*, we send the instance of all intervals and their weights to a weighted interval scheduling algorithm (Step 2). This gives us a set of disjoint intervals in *r*, with, intuitively, a preference of a combination of intervals that are highly supported and covering as much of the read as possible. For each interval, we then send for correction the corresponding segment of the read and all the supporting segments.

The segment correction is performed as follows. We build a partial order alignment graph^41^ from the segments using SPOA^42^ and construct a consensus *c* using the heaviest bundle algorithm^43^ (Step 3). Next, we create a multi-alignment matrix *A* from the pairwise alignments of all the segments to the consensus (we use the method described in ref. ^15^). We generally find this matrix reliable because we expect our segments to be easy to align, since they do not cross exon/intron boundaries.

Given *A* and *c*, we create for every column *j* of *A* a set of trusted contexts and variants, as follows. Let *A*′ correspond to the submatrix of *A* from column *j* − floor(*k*/2) to *j* + floor(*k*/2) (inclusive). A row of *A*′ is classified as a trusted context if it occurs more than a certain number of times in *A*′ (to be made more precise below). The value of this row at the column corresponding to column *j* in *A* is called the trusted variant. This can be a nucleotide or a deletion symbol. We also add as a trusted context the segment of the consensus *c*′ = *c*[*j* − floor(*k*/2): *j* + floor(*k*/2)] and its corresponding variant *c*[*j*].

We decide that a subsegment *b* is a trusted context if it occurs at least max(3, *mT*/min(*ed*(*c*′,*b*), *ed*(*H*(*c*′), *H*(*b*)))) times as a row in *A*′. Here, *m* is the number of segments sent for correction and *T* is a parameter with default value of 0.1. The edit distance term lowers the required threshold for more dissimilar segments to the consensus. This means that lower read depth is required to preserve co-occurring variants (relative to the consensus), and is beneficial under the assumption that co-occurring variants are more common across different reads than co-occurring errors. We use the edit distance between both the original and the homopolymer compressed segments in the denominator to account for the higher number of errors associated with homopolymer lengths. If the strings are identical under homopolymer compression (denominator of 0) the variant (which is a homopolymer length variation) is not considered. Furthermore, we require a variation to be present in at least three reads to be considered.

Once the trusted contexts and variants are established, we error correct the segment of the read *r*, as follows. Let *a* be the row of *A* corresponding to *r*. For every position *j* in *A*, we find the trusted variant whose context has the smallest edit distance to *a*. We replace *a*[*j*] with this variant if it is different than *a*[*j*]. These updates are then projected back onto the sequence of the read. Optionally, we correct the segments in *A*′ belonging to other reads as well (Step 7), described below in heuristic modifications.

#### Heuristic modifications

We refer to the algorithm we have described up to this point as exact. We find that it works fast in practice for small- and medium-size clusters (i.e. for clusters with tens or hundreds of reads). However, for large clusters with thousands of reads this algorithm can be slow, and in this section we describe how we modify it to make it faster. We refer to the modified algorithm as “approximate.” The time bottleneck of the exact algorithm is in Steps 1, 3, and 4. Firstly, we repeatedly call edlib to calculate edit distance for all reads, regions, and identical minimizer combinations. Secondly, we repeatedly do error correction by using spoa and creating the multi-alignment matrix. We take the following action to reduce the running time.

Recall that when error correcting a given read segment *s*, we identify all other read segments *s*′ that support *s* and build an alignment matrix *A*. In the approximate version, we use the opportunity to also error correct all other segments *s*′, using the same alignment matrix *A*. For each *s*′, we store the corrected substring, the support of the instance, as well as the start and end position within the given read as information in a hash table, indexed by the read id. At the time of correcting a read, this hash table will be queried to identify the previously processed regions in this read. The processed regions may overlap. We do not compute the support for these processed regions (Step 1), and instead use the support stored in the hash table. If the processed region is then selected in the scheduling solution, error correction is not done as per Steps 3–4; instead, the corrected substring stored in the hash table is used directly. The approximate algorithm greatly reduces the runtime, as many segments are already computed and corrected in previous iterations.

We also make other heuristic modifications, in addition to the approximate algorithm. We introduce a parameter max_seq_to_spoa to limit the amount of sequences that goes into forming the consensus for very large clusters with spoa (default 200). This reduces runtime without noticeable effect in accuracy. We also mask positional minimizer pairs that contain only A’s in both anchors. This is because many transcripts have a polyA tail, leading the minimizer database to be redundant and repetitive in these regions. Finally, we limit to process max_seq reads at a time within a cluster (default 1000).

As *w* will affect runtime and memory, we set appropriate *w* based on the number of reads in the batch to correct, where *w* is chosen as follows: *w* = *k* + floor{|*C*|/500} where |*C*| is the size of the cluster.

### Experimental

*D. melanogaster* total RNA was isolated from multiple adult W1118 flies of mixed sex according to the protocol outlined in Supplementary Note 4 and sequenced according to the PCS109 protocol (https://community.nanoporetech.com/protocols/cdna-pcr-sequencing_sqk-pcs109/v/PCS_9085_v109_revJ_14Aug2019). Primers were modified so that only the forward primer contained rapid attachment chemistry, resulting in single end adaption of the cDNA representing the 5′ end of the RNA molecule (stranded sequencing). For amplification of the first-strand cDNA, 12 cycles were used and 100 fM of library was loaded onto a FLO-MIN106 flowcell and sequenced for 48 h on the GridION system using MinKnow software v1.14.2. Basecalling was performed in real time using guppy 3.4.8.

Synthetic spike-in transcripts made by Lexogen (SIRV E0): https://www.lexogen.com/store/sirvs SIRV E0 polyA RNA (Lexogen) (1 ng) was used as a template for reverse transcription for use in the PCS109 cDNA by PCR sequencing kit (Oxford Nanopore) following the manufacturer’s instructions (see link above). For amplification of the first-strand cDNA, 12 cycles were used and 100 fM of library was loaded onto a FLO-MIN106 flowcell and sequenced for 48 h on the GridION system. Basecalling was performed in real time using guppy 3.4.8. Only a subset of pass reads with mean base quality larger than 7 were uploaded.

The SIRV and Drosophila data are available on ENA under project accession number PRJEB34849.

### Data analysis

#### Computational processing of the read data

To identify full-length reads among the reads sequenced with ONT we ran pychopper (https://github.com/nanoporetech/pychopper, commit 6dca13d) on Drosophila and SIRV datasets that identifies and removes forward and reverse primers, and splits eventual chimeric reads containing more than one transcript (barcodes in the middle). Only reads deemed to have both a forward and reverse primer are used for downstream analysis. Pychopper was run with default parameters and 50 cores. We kept all reads classified as full-length and longer than 50 nucleotides.

#### Inferring read error rates from alignments

For Drosophila and ONT-old data, where it is unknown which transcripts are sequenced, and novel transcripts compared to annotated transcriptome may be present, we infer read error rates by doing a spliced alignment of reads to the Drosophila reference genome (assembly BDGP6.22) and human reference genome (hg38) using minimap2 with parameters: -w4 -k 13 -ax splice–eqx. The -w 4 is supposed to be more sensitive but higher runtime than the recommended parameters for ONT transcript reads. We then infer insertions, deletions, substitutions from extended cigar strings of the primary alignments (with reads that are unaligned omitted from the analysis). However, we make the following modification not to count small introns as deletions. For a deletion in the cigar string of the genomic alignment, we check whether the coordinates for the deletion matches a previously annotated intron from an annotated transcriptome database. We use Ensembl release 97 annotated on assembly BDGP6.22 for the Drosophila data, and Ensembl release 101 annotated on hg38 for the ONT-old data. If the deletion start and stop coordinates matches the intron annotation, we do not count it towards a deletion. We then say that for a read, the "% difference to the genome" is the total number of insertions, deletions, and substitutions divided by the alignment length, which is the total number of insertions, deletions, substitutions and matches.

For SIRV and simulated data, where we have the true transcripts present in the sequencing material, we infer read error rates by aligning reads to the transcriptome consisting of 68 synthetic transcripts using minimap2 with parameters -w1 -k 8 -a–eqx. We infer insertions, deletions, substitutions from the extended cigar strings of the alignments, but do not make the modification for intron deletions as we did for genomic alignments. The mismatch rate is computed as the sum of insertions, deletions, and substitutions divided by the alignment length.

#### SIRV subsampling experiments

The 68 SIRV transcripts contain five transcripts that are perfect substrings of other larger transcripts. These substring transcripts confound the alignments of the reads and the error rate calculations, so we filtered them out for the analyses we did with subsampled SIRV data. We aligned the 1,514,274 full-length SIRV reads to the remaining 63 SIRV transcripts.

For the experiment where we investigated error rates as a function of read depth, we ran 100 experiments, with 10 replicates in each. For each value of *y* between 1 and 100, we subsampled *y* aligned reads from each transcript. This resulted in a dataset of 63·*y* reads with an expected read depth of *y*. For each *y*, we did 10 replicates, to alleviate sampling variation. This gave a total of 1000 simulations.

For the experiment where we investigated error rates as a function of both read depth and number of isoforms, we ran 60 experiments with varying numbers of isoforms and read depth, with 10 replicates in each experiment. Concretely, for each of the three SIRV genes, for each *y* ∈ {1,4,8}, and for each value of *x* between 1 and 20, an experiment was performed. In the experiment, we randomly picked *y* isoforms of the given gene and, for each isoform, randomly sampled *x* reads from the reads aligning to that isoform. Each experiment was repeated 10 times to obtain replicates. Each set of replicates resulted in a controlled dataset with 10 replicates and each replicate with 3 · *x* · *y* reads and a depth of *x* reads per transcript.

For each simulation in the above described experiments, we clustered the reads with isONclust (git commit 5b969b6d) with default parameters for ONT data. Then, we ran isONcorrect on the clusters, using the default parameters *k* = 9, *x*_min_ = 2*k*, *x*_max_ = 80, and exact_instance_limit 50, that computes exact mode for clusters smaller than 50 reads.

#### Splice sites

To classify Drosophila reads, we use minimap2 to align reads to the Drosophila reference genome. We classify as a splice site everything that minimap2 flags as an intron location or any deletions (relative to the reference) whose start and stop sites match a true intron annotation in the ENSEMBL annotations. The second condition is necessary not to count small introns that are preserved in the reads but flagged as deletions in the alignment due to their small size (we observed introns as small as only two bases). We then match the splice sites of the alignments to existing Drosophila annotations and classify the transcripts according to the four categories defined by Tardaguila et al.^28^ as follows. A transcript is an FSM if all its start and stop splice sites are in the database annotation and the particular combination of start and stop splice sites matches that of a known transcript; incomplete splice match (ISM) if all its start and stop splice sites are in the database annotation and they match match a consecutive subset of start and stop splice sites of an annotated transcript; NIC if all the individual start and stop splice sites are in the database annotation but they create a new combination of start and stop splice sites, or; novel-not-in-catalog (NNC) if the transcript has at least one splice site that is not in the database.

#### Effect of parameters and heuristics experiments

First we aligned all SIRV reads to the 68 distinct transcripts (we observed the coverage shown in Supplementary Fig. 11). We then subsampled, without replacement, 3, 5, 10, and 20 reads that had unambiguous primary alignments from four randomly selected transcripts, with the requirement that the transcript had more unambiguous primary alignments than the required subsample size. We run isONcorrect on these datasets and measure the error rate of the corrected reads using both exact and approximate correction. We repeat the above experiment 10 times to alleviate variation from picking specific transcripts and reads.

### Reporting summary

Further information on research design is available in the Nature Research Reporting Summary linked to this article.

## Supplementary information

Supplementary Information

Reporting Summary

## Data Availability

The SIRV and Drosophila data are available on ENA under project accession number PRJEB34849. Drosophila reference genome (assembly BDGP6.22) was downloaded at ftp://ftp.ensembl.org/pub/release-97/fasta/drosophila_melanogaster/dna/Drosophila_melanogaster.BDGP6.22.dna.toplevel.fa.gz. The human reference genome (hg38) was downloaded from ftp://ftp.ensembl.org/pub/release-101/fasta/homo_sapiens/dna/Homo_sapiens.GRCh38.dna.primary_assembly.fa.gz. We use Ensembl release 97 annotated on assembly BDGP6.22 for the Drosophila data, downloaded from ftp://ftp.ensembl.org/pub/release-97/gtf/drosophila_melanogaster/Drosophila_melanogaster.BDGP6.22.97.gtf.gz). We used Ensembl release 101 on hg38 for the ONT-old data, downloaded from ftp://ftp.ensembl.org/pub/release-101/gtf/homo_sapiens/Homo_sapiens.GRCh38.101.gtf.gz. The SIRV genes and gene annotations were downloaded from https://www.lexogen.com/wp-content/uploads/2018/08/SIRV_Set1_Lot00141_Sequences_170612a-ZIP.zip. The ONT-old dataset was downloaded from https://s3.amazonaws.com/nanopore-human-wgs/rna/fastq/Bham_Run1_20171115_1D.pass.dedup.fastq.

## Code availability

All scripts used for simulating datasets as well as to run the evaluation are found at https://github.com/ksahlin/isONcorrect. Specifically, the evaluation pipeline for simulated data is found under https://github.com/ksahlin/isONcorrect/tree/master/evaluation_sim, for SIRV data under https://github.com/ksahlin/isONcorrect/tree/master/evaluation_sirv, and for biological data under https://github.com/ksahlin/isONcorrect/tree/master/evaluation. The source code of isONcorrect is available at https://github.com/ksahlin/isONcorrect.
