# Author Correction: Error correction enables use of Oxford Nanopore technology for reference-free transcriptome analysis

**DOI:** 10.1038/s41467-021-21424-9

**Published:** 2021-02-08

**Authors:** Kristoffer Sahlin, Paul Medvedev

**Affiliations:** 1 Department of Mathematics, Science for Life Laboratory, Stockholm University, 106 91 Stockholm, Sweden; 2 Department of Computer Science and Engineering, The Pennsylvania State University, University Park, PA USA; 3 Department of Biochemistry and Molecular Biology, The Pennsylvania State University, University Park, PA USA; 4 Center for Computational Biology and Bioinformatics, The Pennsylvania State University, University Park, PA USA

**Keywords:** RNA sequencing, Data processing, Transcriptomics

Correction to: *Nature Communications* 10.1038/s41467-020-20340-8, published online 4 January 2021.

The original version of this Article included second and third authors who wish to be removed from authorship and instead mentioned in the Acknowledgements. Consequently, the author affiliation for these authors has been removed and the equal contributions statement has been removed. The author affiliations have changed from “^1^Department of Mathematics, Science for Life Laboratory, Stockholm University, 106 91 Stockholm, Sweden. ^2^Oxford Nanopore Technologies Ltd, Oxford, UK. ^3^Department of Computer Science and Engineering, The Pennsylvania State University, University Park, PA, USA. ^4^Department of Biochemistry and Molecular Biology, The Pennsylvania State University, University Park, PA, USA. ^5^Center for Computational Biology and Bioinformatics, The Pennsylvania State University, University Park, PA, USA” to read “^1^Department of Mathematics, Science for Life Laboratory, Stockholm University, 106 91 Stockholm, Sweden. ^2^Department of Computer Science and Engineering, The Pennsylvania State University, University Park, PA, USA. ^3^Department of Biochemistry and Molecular Biology, The Pennsylvania State University, University Park, PA, USA. ^4^Center for Computational Biology and Bioinformatics, The Pennsylvania State University, University Park, PA, USA”. The following was added to the Acknowledgements: “We want to thank Botond Sipos for several substantially helpful discussions regarding data analysis, and Philip L. James for producing the biological sequencing data”. The original version of the Author contribution statement”, which read: “K.S. and P.M. conceived the project. K.S. designed the algorithm with input from P.M. and B.S. K.S. implemented the algorithm. K.S. and P.M. designed the evaluation and experiments. K.S. implemented the evaluation. K.S. and P.M. analyzed the results with input from B.S. B.S. implemented the preprocessing tool pychopper with input from K.S. P.L.J. performed the wet-lab experiments. K.S. and P.M. drafted the paper with contributions from B.S. All authors critically revised the text”, has been amended to read: “K.S. and P.M. conceived the project, designed the evaluation and experiments, analyzed the results, and wrote the paper. K.S. designed the algorithm with input from P.M. K.S. implemented the algorithm and evaluation. All authors critically revised the text”. The Competing interest statement, which originally read: “Data generation was funded by Oxford Nanopore Technologies (ONT). B.S. and P.J. are ONT employees and stock option holders. The remaining authors declare no competing interests”, has been amended to read: “Data generation was funded by Oxford Nanopore Technologies (ONT). The remaining authors declare no additional competing interests”.

This has been corrected in both the PDF and HTML versions of the Article.

