# Supplementary material for: Error correction enables use of Oxford Nanopore technology for reference-free transcriptome analysis
Source: Nat Commun. 2021 Jan 4;12:2. doi: 10.1038/s41467-020-20340-8 (PMC7782715; doi:10.1038/s41467-020-20340-8)
Supplement: Supplementary file 1 — Supplementary Information [file 41467_2020_20340_MOESM1_ESM.pdf]

Supplementary Figures and Notes

Error correction enables use of Oxford Nanopore technology for reference-free transcriptome analysis

Kristoffer Sahlin[4,5]*, Botond Sipos[6]*, Phillip L James[6], Paul Medvedev[1,2,3]†

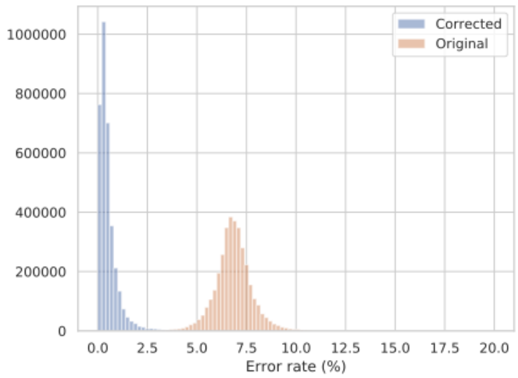

Supplementary Figure 1: Error rates of the SIM-full reads before and after error correction by isONcorrect.

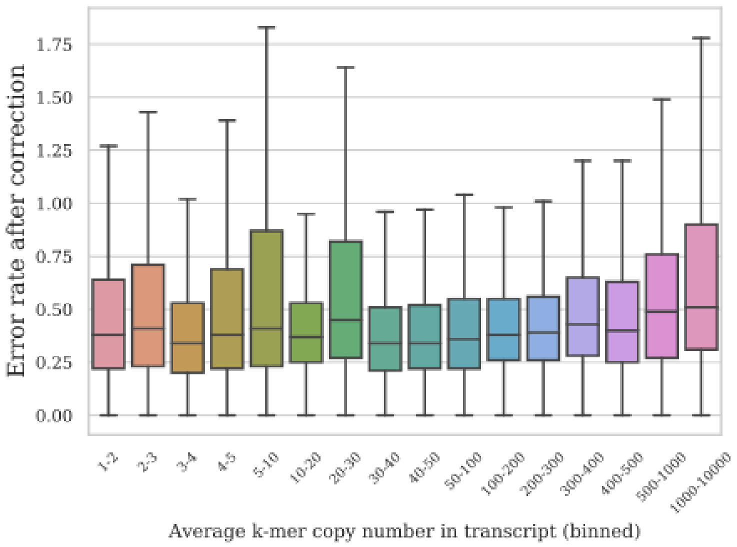

Supplementary Figure 2. Error rates of reads after correction broken down per average mappability of transcripts that the read originates from. The average mappability is derived from a kmer size of 31. The boxplot shows the median average mappability, the colored boxes show the interquartile range (middle 50%), the thinner black line is the 95% confidence interval. The number of reads (N) for the boxes on the x-axis are, for each x-value; 2,927,471, 63,693, 47,367, 13,990, 57,065, 38,474, 20,366, 21,268, 21,468, 66,660, 94,248, 45,377, 19,350, 16,406, 35,298, 11,499, respectively.

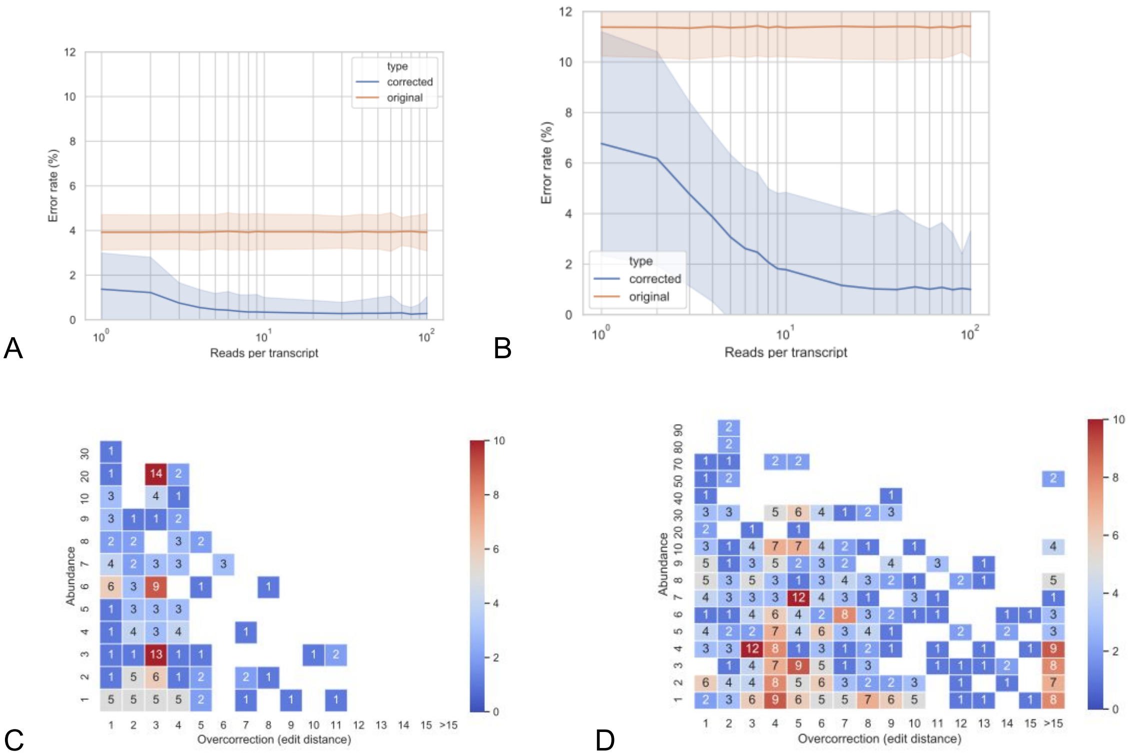

Supplementary Figure 3. The effect of varying pre-correction error rates on isONcorrect. Effect of read depth on error rate for the (A) SIM-ca-4 dataset and (B) SIM-ca-11 dataset. Overcorrection in the (C) SIM-ca-4 dataset and (D) SIM-ca-11 dataset. In (A) and (B) the shaded areas show the standard deviation (SD) of the error rates.

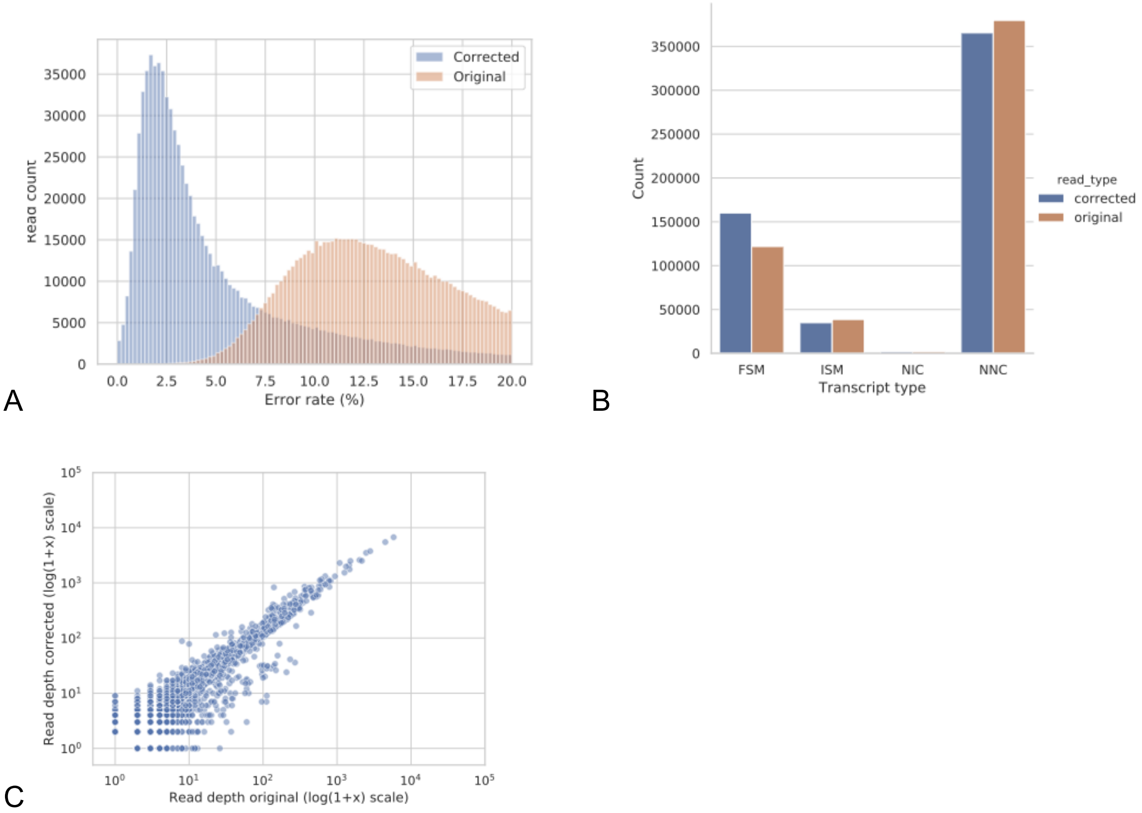

Supplementary Figure 4. isONcorrect performance on the ONT-old dataset. (A) Error rates of the ONT-old reads before and after error correction by isONcorrect. (B) Total number of reads classified per splice site category. (C) For each transcript in the reference, we measure the number of reads aligning to it as a FSM, before and after error correction. Each dot represents a distinct transcript with at least one FSM in either the original or corrected reads.

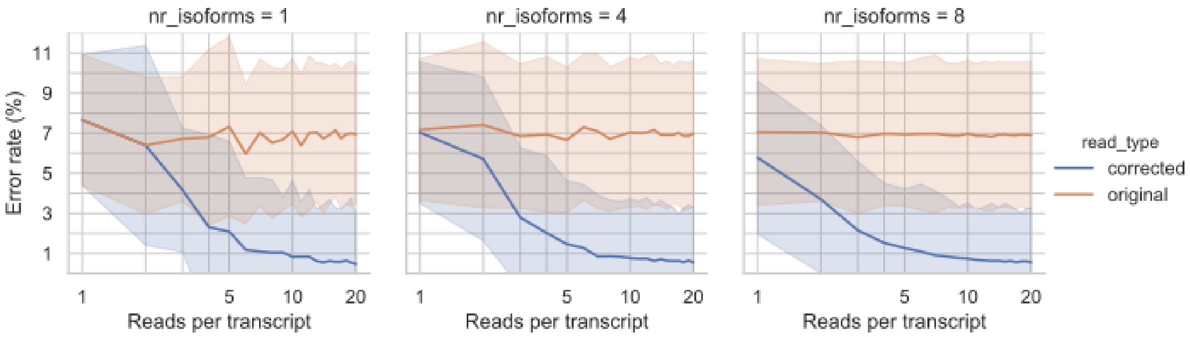

Supplementary Figure 5. Effect of the number of isoforms on error rates, using subsampled SIRV datasets with varying numbers of isoforms and read depths. The shaded areas show the standard deviation of the data, aggregated over each x-value.

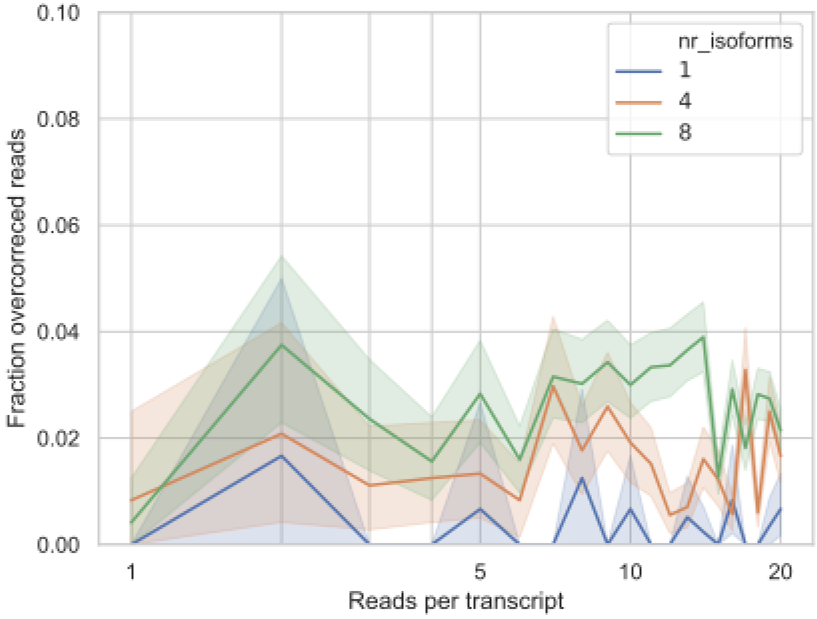

Supplementary Figure 6. Effect of the number of isoforms on overcorrection, using the same subsampled SIRV datasets as in Fig. S4. The y-axis shows the percentage of overcorrected reads. The shaded areas show the standard deviation of the data, aggregated over each x-value.

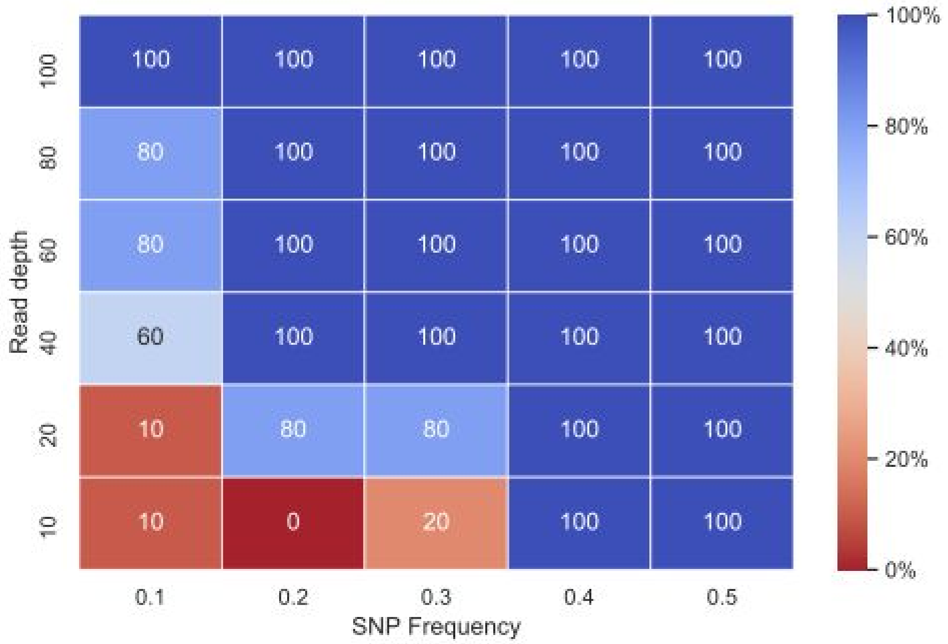

Supplementary Figure 7: IsONcorrect's ability to retain a minor-allele SNP in the case of allele-specific expression. The percentage of replicate experiments where the minor allele SNP is preserved in the dataset (value in each cell) as a function of the minor allele frequency (x-axis) and the total number of reads (y-axis).

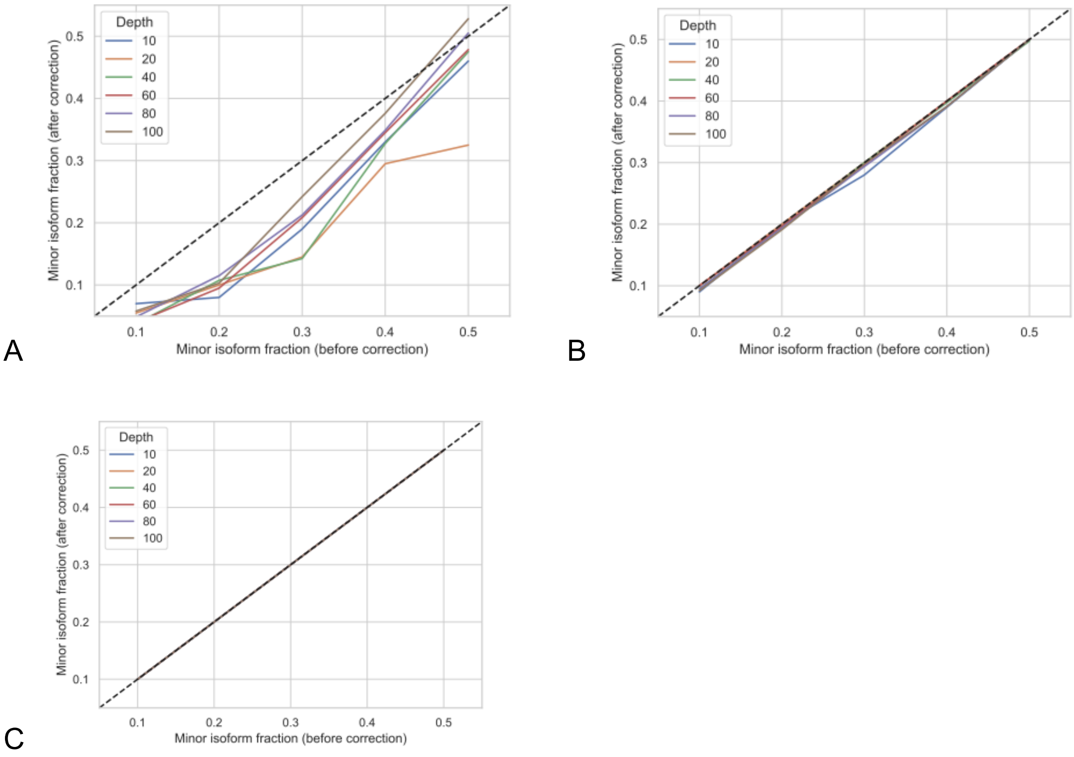

Supplementary Figure 8. Perturbance of isoform abundance after error correction with isONcorrect using simulated data for a (A) 5bp exon, (B) 10bp exon, (C) 20bp exon.

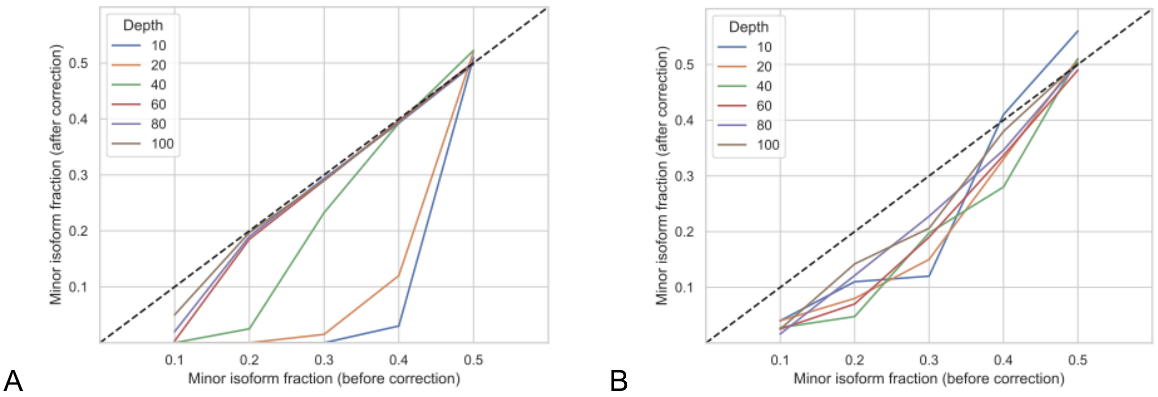

Supplementary Figure 9. Perturbance of isoform abundance after error correction with isONcorrect using SIRV data for a (A) 6bp exon and (B) 14bp exon.

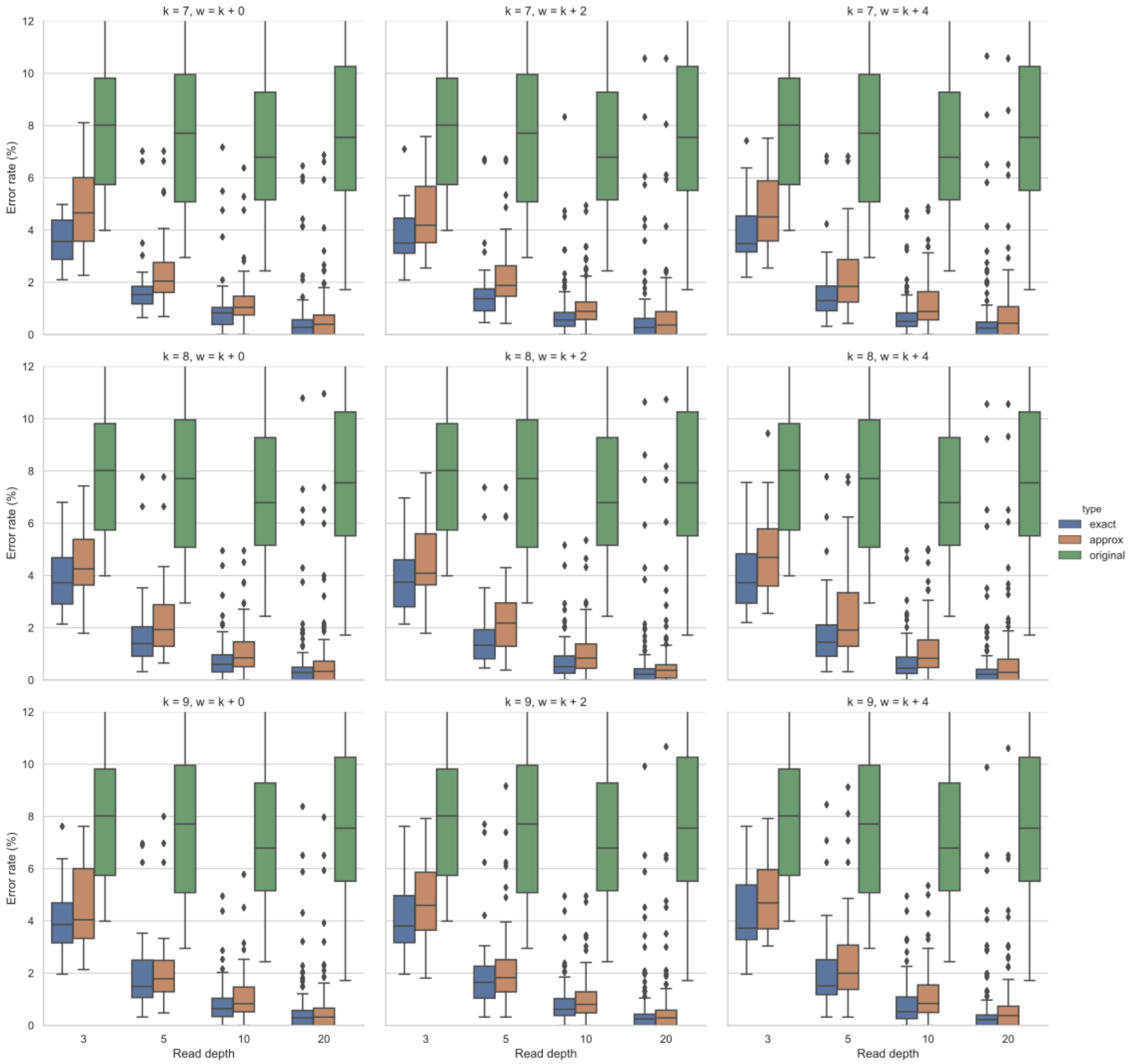

Supplementary Figure 10. The effect on the error rate of parameters k and w, read depth, and the heuristic approximation algorithm. Each panel is labeled with a fixed value of k and w. The boxplot shows the median error rate, the colored boxes show the interquartile range (middle 50%), the thinner black line is the 95% confidence interval, and the diamonds show the outliers. For each value of 3, 5, 10, and 20 on the x-axis, the total number of reads in each box (N) is 30, 50, 100, and 200 for (from 10 replicate experiments for each parameter setting).

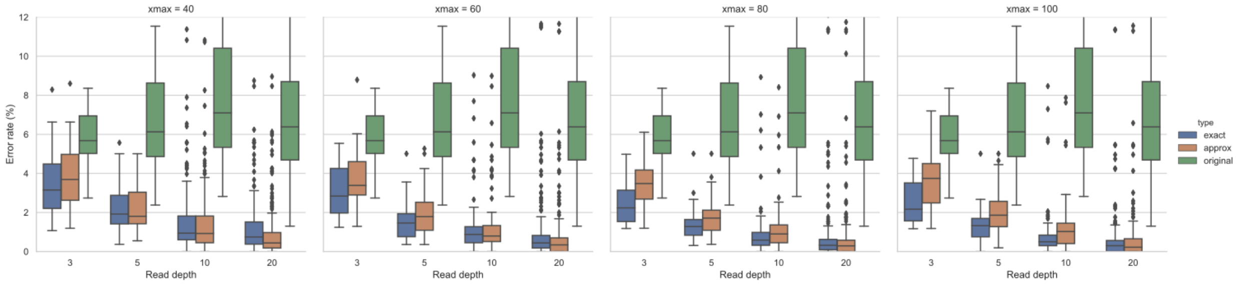

Supplementary Figure 11. The effect on the error rate of the maximum anchor distance x_{max} , read depth, and the heuristic approximation algorithm. Each panel is labeled with a fixed value of x_{max} . The value of k and w is fixed to 9 in these experiments. The boxplot shows the median error rate, the colored boxes show the interquartile range (middle 50%), the thinner black line is the 95% confidence interval, and the diamonds show the outliers. For each value of 3, 5, 10, and 20 on the x-axis, the total number of reads in each box (N) is 30, 50, 100, and 200 for (from 10 replicate experiments for each parameter setting).

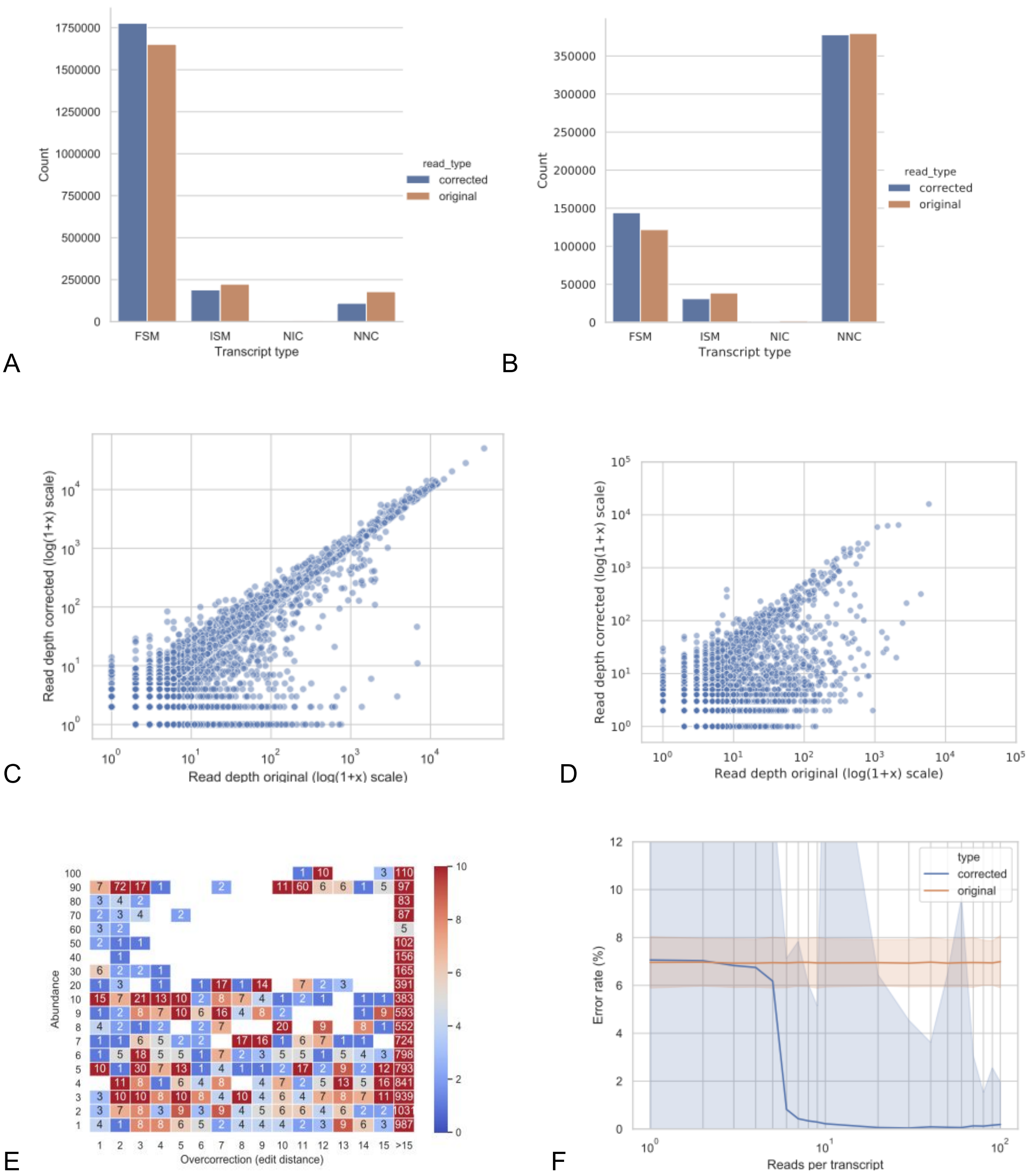

Supplementary Figure 12. Splice site accuracy and overcorrection with RATTLE. Total number of reads classified per splice site category, using the terminology of [1] in the Drosophila data (A) and ONT-old data (B). In (C) and (D) we measure the number of reads aligning as an FSM to a transcript in the Drosophila and human reference, respectively, before and after error correction. Each dot represents a distinct transcript with at least one FSM in either the original or corrected reads. (E) The effect of overcorrection in the SIM-ca data using RATTLE. We bin each overcorrected read according to the abundance of its true transcript (y-axis) and its overcorrection distance (x-axis). Each cell shows the number of reads in the bin. (F) The median error rate of the SIM-ca dataset based on controlled read depth of the transcript. The shaded area shows the standard deviation (SD) of the error rates, aggregated over each x-value.

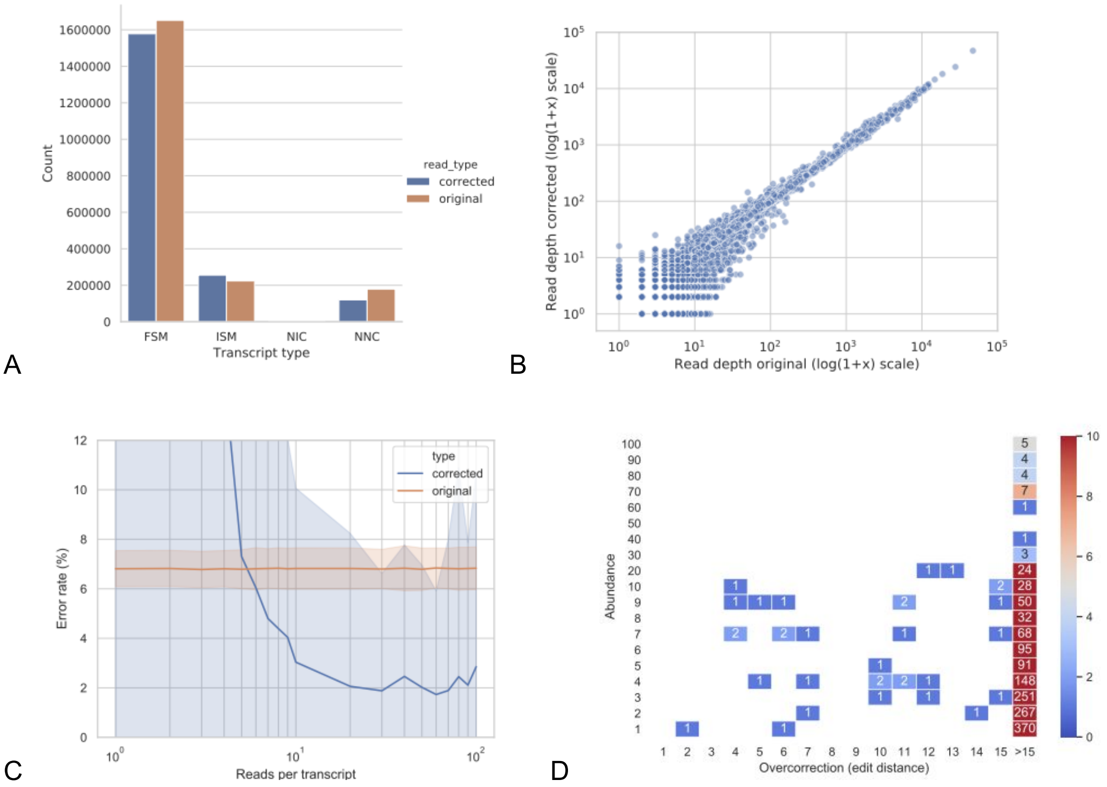

Supplementary Figure 13. Canu error correction performance. (A) Total number of reads classified per splice site category on the *Drosophila* dataset. (B) For each transcript in the *Drosophila* reference, we measure the number of reads aligning to it as a FSM, before and after error correction. Each dot represents a distinct transcript with at least one FSM in either the original or corrected reads. (C) The median error rate of the SIM-ca dataset based on controlled read depth of the transcript. The shaded area shows the standard deviation (SD) of the error rates, aggregated over each x-value. (D) The effect of overcorrection in the SIM-ca data using canu. We bin each overcorrected read according to the abundance of its true transcript (y-axis) and its overcorrection distance (x-axis). Each cell shows the number of reads in the bin.

Supplementary Note 1: Simulation design

Before implementing our own transcriptomic read simulator, we explored the possibility of using already existing read simulators such as NanoSim [2], DeepSimulator [3], SimLord [4], and SNaReSim [5]. However, they are all genomic read simulators and cannot easily be modified to simulate full-length transcript reads and to output quality values, as well as to sample reads from transcripts at controlled abundances.

We downloaded 10,384 ENSEMBL transcripts from human chromosome 6 and filtered them down to 10,367 distinct transcripts (distinct meaning they are not identical sequences). We chose chromosome 6 because it harbors transcripts from difficult instances such as the highly polymorphic HLA loci. For each transcript, we assign an abundance from the set $A = \{1, 2, ..., 10, 20, 30, .., 100\}$, with the probability of an abundance a being $p_a = \frac{1}{a \sum_{b \in A} 1/b} \approx .32a^{-1}$.

The effect of this simulation design is that most transcripts (32%) are expected to have an abundance of 1, and fewest transcripts (0.32%) are expected to have an abundance of 100. We use this weighted abundance in both our simulated experiments. In the full chr6 experiment we generate 3.5M reads randomly from this relative abundance distribution. For the controlled subsampling experiments, we generate precisely a full length reads from a transcript with abundance a.

To simulate a read we pick a quality value q uniformly at random over each base pair in the transcript from a set Q of quality values. The base is assigned the Phred score corresponding to q. Then, with probability q, we make the base erroneous. We simulate the error type as either deletion, substitution, or insertion with probabilities of 0.45, 0.35, and 0.2, respectively. These values were chosen to reflect the error profile that we observed in our real data. In the SIM-full and SIM-ca experiments we set $Q = \{0.85, 0.875, 0.9, 0.92, 0.96, 0.98, 0.99, 0.995\}$ to obtain a median error rate of ~7% as is seen in biological data. When we investigated error rates of ~3.94% (SIM-ca-4) and ~11.40% (SIM-ca-11), we used the sets $\{0.9, 0.95, 0.96, 0.98, 0.99, 0.995\}$ and $\{0.75, 0.85, 0.875, 0.91, 0.95, 0.98\}$, respectively. These contained 59,572 and 60,336 simulated reads, respectively.

If the error type is a substitution, we uniformly replace the sequenced base with one of the three alternate bases and set the base's Phred quality score based on q. If the error is an insertion, we enter an insertion state where we generate an inserted base with probability 0.3, or exit this state otherwise. Thus, multiple bases are more likely to be inserted consecutively. Inserted bases are drawn uniformly at random over A, C, G, and T. As for the quality values in an insertion, the current base and the first insertion will have quality value corresponding to q and the rest of the inserted bases, if any, will have a Phred quality score corresponding to 0.7, so that the error rate roughly matches the error rate in the Phred quality values. If the error is a

deletion, the base is simply omitted, however, the quality score will be propagated to the next base, if the next base is simulated to be correct.

Supplementary Note 2: Error rate in repetitive regions

A fraction of transcripts may come from multi-gene copies, or contain regions that are highly repetitive with respect to the transcriptome or genome because of satellite DNA, or LINE repeats. We used our human simulated dataset (SIM-full) to investigate whether correction performance was affected by repetitive regions in the transcripts. To study this we computed the average copy number of each kmer in the reference genome (Hg38) for each transcript (using k-mer size of 31) for the SIM-full dataset where we have the true annotations. We then plotted the error rates of reads after correction for the reads in SIM-full binned according to the average k-mer copy number (Supplementary Fig. 2). We do not observe any clear dependence of the post-correction error rate on the repetitiveness of the gene sequence.

While it is expected that difficulties in multi-mapping reads would make reference-based correction difficult, a reference-agnostic approach does not suffer from trusting genome mis-alignments. In fact, any gene with multiple isoforms will behave like a repetitive region to a transcriptomic error correction algorithm, and hence such algorithms are by necessity designed to avoid being misguided by such similarities.

Supplementary Note 3: Evaluation of CONSENT and canu

We used the same machine to evaluate CONSENT[6] and canu[7] as we evaluated isONcorrect on. The machine was an x86_64 system running Linux v3.2.0-4-amd64 and equipped with 32 2-threaded cores and 512 GB RAM. We ran CONSENT using parameters --type ONT and --nproc 60. CONSENT returned an error on the SIRV dataset after approximately two days and on the Drosophila dataset after approximately 31 days. On the SIM-full dataset, we cancelled the job after 216 hours (9 days) without any output. These results are not surprising since CONSENT was designed for genomic, rather than transcriptomic, data.

We ran canu using parameters, -correct, useGrid=false corOutCoverage=all minReadLength=100 minOverlapLength=100 -maxThreads=60 -maxMemory=480g corOverlapper=minimap -nanopore. We also set the genomeSize parameter (which is supposed to be the estimated genome size) to the size of the transcriptome as performed in [8] and inspired by discussions at https://github.com/marbl/canu/issues/641. Specifically, we set it to 30m, 18m, and 75k for Drosophila, SIM-full, and SIRV, respectively. On the SIM-ca dataset, the average coverage per transcript is lower than the canu minimum suggested coverage (10x). As expected, canu returned an error on this dataset related to coverage and we therefore (as suggested in the output error message) reduced genomeSize to 1m.

Canu produced a post-correction error rate of 2.0%, 2.7%, and 1.9% for the SIRV, Drosophila, and the SIM-full dataset, respectively. This is significantly higher than both isONcorrect and RATTLE[9] (Table 2). Canu also produced 266,659 (8%) reads with a higher number of errors after correction, on the SIM-full dataset, compared to only 1,578 reads for isONcorrect (Table 2). The number of reads with more errors after correction was 4,622 and 8,292 for the SIRV and Drosophila datasets, respectively, which is comparable to isONcorrect (Table 2). However, as we do not have the true annotations for the non-simulated datasets, this analysis does not include reads that have been overcorrected into another isoform.

We also studied the effect of error correction to alterations in splice structure using the *Drosophila* data. As opposed to isONcorrect and RATTLE, canu decreased the number of reads fully matching an annotated transcript (FSM) on Drosophila (Supplementary Fig. 13A), and we also observed significantly less reference transcripts having at least one canu corrected FSM read (11,127) than having at least one original FSM read (13,062). This corresponds to a 14.8% reduction for Drosophila compared to only a 0.6% reduction for isONcorrect. Specifically, for Drosophila canu lost 2,023 and gained 80 transcripts with at least one FSM, while isONcorrect lost 180 and gained 100 transcripts. As for isONcorrect, mostly transcripts with few FSM reads disappeared after correction with canu (Supplementary Fig. 13B).

We further investigated canu's performance on the SIM-ca dataset with variable abundances (Supplementary Fig. 13C-D). The results on this dataset shows that variable coverage is a challenge for genomic error correctors that assume uniform coverage. Canu's post-correction error rate was 3.8%, which is substantially higher than isONcorrect and RATTLE (0.6% and 0.4%, respectively). The median error rate only improved for a coverage above 10 (Supplementary Fig. 13C). Furthermore, canu overcorrected 1,482 reads in this dataset compared to 214 (isONcorrect) and 6,541 (RATTLE) on this dataset and these overcorrected reads were mostly substantial overcorrections (Supplementary Fig. 13D) and were found throughout different abundances.

The relatively high post-correction error rates, together with substantial overcorrection and the inability to correct reads at various coverages, we conclude that canu is not suitable for transcriptomic error correction. As with CONSENT, this is not surprising since canu was not designed with this type of data in mind.

Supplementary Note 4: Protocol for generating total RNA

1) Add 500 µl of TRIzol to a 1.5 ml Eppendorf with 50 mg of flies and homogenise with a disposable cell pestle.
2) Add 500 µl of TRIzol and invert multiple times to mix.
3) Incubate at room temperature for 15 minutes.
4) Centrifuge at 4oC for 10 minutes at 12000 x g.
5) Transfer supernatant to a new 1.5 ml Eppendorf.
6) Add 200 µl of chloroform and invert to mix.
7) Incubate at room-temperature for 10 minutes.
8) Centrifuge at 4oC during 15 minutes at 10000 x g.
9) Transfer supernatant to a new 1.5 ml Eppendorf tube and add 500 µl of ice-cold isopropanol.
10) Invert several times to mix and incubate 15 minutes at room temperature.
11) Centrifuge at 4oC during 10 minutes at 10000 x g.
12) Discard supernatant and add 1000 ul of 70% ice-cold ethanol.
13) Invert several times to wash the pellet.
14) Centrifuge at 4oC during 5 minutes at 10000 x g.
15) Discard the ethanol and use a sterile wipe to absorb the remaining ethanol from the tube walls.
16) Elute in 200 µl of TE. 150 fM of total RNA was used as a template for reverse transcription for use in the PCS-109 cDNA by PCR sequencing kit (Oxford Nanopore) Following the manufacturer's instructions (https://community.nanoporetech.com/protocols/cdna-pcr-sequencing_sqk-pcs109/v/PCS_9085_v109_revJ_14Aug2019).

References

1. Tardaguila, M. *et al.* SQANTI: extensive characterization of long-read transcript sequences for quality control in full-length transcriptome identification and quantification. *Genome Research* vol. 28 396–411 (2018).
2. Yang, C., Chu, J., Warren, R. L. & Birol, I. NanoSim: nanopore sequence read simulator based on statistical characterization. *Gigascience* **6**, 1–6 (2017).
3. Li, Y. *et al.* DeepSimulator: a deep simulator for Nanopore sequencing. *Bioinformatics* **34**, 2899–2908 (2018).
4. Stöcker, B. K., Köster, J. & Rahmann, S. SimLoRD: Simulation of Long Read Data. *Bioinformatics* vol. 32 2704–2706 (2016).
5. SNaReSim: Synthetic Nanopore Read Simulator - IEEE Conference Publication. https://ieeexplore.ieee.org/document/8031171.
6. Morisse, P., Marchet, C., Limasset, A., Lecroq, T. & Lefebvre, A. CONSENT: Scalable self-correction of long reads with multiple sequence alignment. bioRxiv, feb 2019. doi: 10.1101/546630. *URL https://doi. org/10. 1101/546630*.
7. Koren, S. *et al.* Canu: scalable and accurate long-read assembly via adaptive k-mer weighting and repeat separation. *Genome Res.* **27**, 722–736 (2017).
8. Lima, L. *et al.* Comparative assessment of long-read error correction software applied to Nanopore RNA-sequencing data. *Brief. Bioinform.* **21**, 1164–1181 (2020).
9. de la Rubia, I. *et al.* Reference-free reconstruction and quantification of transcriptomes from long-read sequencing. 2020.02.08.939942 (2020) doi:10.1101/2020.02.08.939942.
