# Supplementary material for: Error correction enables use of Oxford Nanopore technology for reference-free transcriptome analysis
Source: Nat Commun. 2021 Jan 4;12:2. doi: 10.1038/s41467-020-20340-8 (PMC7782715; doi:10.1038/s41467-020-20340-8)
Supplement: Supplementary file 2 — Reporting Summary [file 41467_2020_20340_MOESM2_ESM.pdf]

nature research

Corresponding author(s): Paul Medvedev

Last updated by author(s): Oct 15, 2020

Reporting Summary

Nature Research wishes to improve the reproducibility of the work that we publish. This form provides structure for consistency and transparency in reporting. For further information on Nature Research policies, see Authors & Referees and the Editorial Policy Checklist.

Statistics

For all statistical analyses, confirm that the following items are present in the figure legend, table legend, main text, or Methods section.

n/a	Confirmed	
☐	☒	The exact sample size (*n*) for each experimental group/condition, given as a discrete number and unit of measurement
☒	☐	A statement on whether measurements were taken from distinct samples or whether the same sample was measured repeatedly
☒	☐	The statistical test(s) used AND whether they are one- or two-sided<br>*Only common tests should be described solely by name; describe more complex techniques in the Methods section.*
☒	☐	A description of all covariates tested
☒	☐	A description of any assumptions or corrections, such as tests of normality and adjustment for multiple comparisons
☐	☒	A full description of the statistical parameters including central tendency (e.g. means) or other basic estimates (e.g. regression coefficient) AND variation (e.g. standard deviation) or associated estimates of uncertainty (e.g. confidence intervals)
☒	☐	For null hypothesis testing, the test statistic (e.g. *F*, *t*, *r*) with confidence intervals, effect sizes, degrees of freedom and *P* value noted<br>*Give P values as exact values whenever suitable.*
☒	☐	For Bayesian analysis, information on the choice of priors and Markov chain Monte Carlo settings
☒	☐	For hierarchical and complex designs, identification of the appropriate level for tests and full reporting of outcomes
☒	☐	Estimates of effect sizes (e.g. Cohen's *d*, Pearson's *r*), indicating how they were calculated

Our web collection on statistics for biologists contains articles on many of the points above.

Software and code

Policy information about availability of computer code

Data collection	Data was collected using MinKnow v1.14.2
Data analysis	Custom software was developed and is available on GitHub https://github.com/ksahlin/isONcorrect. Basecalling was performed in real time using guppy 3.4.8 (freely available at https://ontpipeline2.readthedocs.io/en/latest/GetStarted.html). pychopper was used to trim the reads from adapters (freely available at https://github.com/nanoporetech/pychopper). Pychopper version used was commit ID 6dca13d in GitHub

For manuscripts utilizing custom algorithms or software that are central to the research but not yet described in published literature, software must be made available to editors/reviewers. We strongly encourage code deposition in a community repository (e.g. GitHub). See the Nature Research guidelines for submitting code & software for further information.

Data

Policy information about availability of data

All manuscripts must include a data availability statement. This statement should provide the following information, where applicable:

- Accession codes, unique identifiers, or web links for publicly available datasets
- A list of figures that have associated raw data
- A description of any restrictions on data availability

All sequence data deposited under ENA study accession PRJEB34849.
Drosophila reference genome (assembly BDGP6.22) was downloaded at ftp://ftp.ensembl.org/pub/release-97/fasta/drosophila_melanogaster/dna/Drosophila_melanogaster.BDGP6.22.dna.toplevel.fa.gz. The human reference genome (hg38) was downloaded from ftp://ftp.ensembl.org/pub/release-101/fasta/homo_sapiens/dna/Homo_sapiens.GRCh38.dna.primary_assembly.fa.gz. We use Ensembl release 97 annotated on assembly BDGP6.22 for the Drosophila data, downloaded from ftp://ftp.ensembl.org/pub/release-97/gtf/drosophila_melanogaster/Drosophila_melanogaster.BDGP6.22.97.gtf.gz). We used Ensembl release 101 on hg38 for the ONT-old data, downloaded from ftp://ftp.ensembl.org/pub/release-101/gtf/homo_sapiens/Homo_sapiens.GRCh38.101.gtf.gz. The SIRV genes and gene annotations were downloaded from https://www.lexogen.com/wp-content/uploads/2018/08/SIRV_Set1_Lot00141_Sequences_170612a-ZIP.zip. The

ONT-old dataset was downloaded from https://s3.amazonaws.com/nanopore-human-wgs/rna/fastq/Bham_Run1_20171115_1D.pass.dedup.fastq

Field-specific reporting

Please select the one below that is the best fit for your research. If you are not sure, read the appropriate sections before making your selection.

☒ Life sciences ☐ Behavioural & social sciences ☐ Ecological, evolutionary & environmental sciences

For a reference copy of the document with all sections, see nature.com/documents/nr-reporting-summary-flat.pdf

Life sciences study design

All studies must disclose on these points even when the disclosure is negative.

Sample size	We sequenced one Drosophila individual and one synthetic sample. Since this study focused on computational performance, rather than any novel conclusions about the individuals or species, only one sample was used.
Data exclusions	No data was excluded from the analysis
Replication	We did not replicate any experiment outside of what is mentioned in the experiment design and communicated in the figures. Where applicable, the experiments are instead designed to account for variability.
Randomization	N/A: There were no experimental group.
Blinding	Blinding was not relevant to this study because there were no participants and clinical study was performed.

Reporting for specific materials, systems and methods

We require information from authors about some types of materials, experimental systems and methods used in many studies. Here, indicate whether each material, system or method listed is relevant to your study. If you are not sure if a list item applies to your research, read the appropriate section before selecting a response.

Materials & experimental systems

n/a	Involved in the study	
☒	☐	Antibodies
☒	☐	Eukaryotic cell lines
☒	☐	Palaeontology
☐	☒	Animals and other organisms
☒	☐	Human research participants
☒	☐	Clinical data

Methods

n/a	Involved in the study	
☒	☐	ChIP-seq
☒	☐	Flow cytometry
☒	☐	MRI-based neuroimaging

Animals and other organisms

Policy information about studies involving animals; ARRIVE guidelines recommended for reporting animal research

Laboratory animals	D. melanogaster total RNA was isolated from multiple adult W1118 flies of mixed sex.
Wild animals	None
Field-collected samples	None
Ethics oversight	No laboratory animals were used. The study required an ethics approval.

Note that full information on the approval of the study protocol must also be provided in the manuscript.
